# Application of mutational profiling: New functional analyses reveal the tRNA recognition mechanism of tRNA m^1^A22 methyltransferase

**DOI:** 10.1016/j.jbc.2022.102759

**Published:** 2022-12-01

**Authors:** Ryota Yamagami, Hiroyuki Hori

**Affiliations:** Department of Materials Science and Biotechnology, Graduate School of Science and Engineering, Ehime University, Matsuyama, Ehime, Japan

**Keywords:** transfer RNA, RNA methyltransferase, RNA modification, reverse transcription, high-throughput screening, RNA–protein interaction, MaP, Mutational Profiling, RT, reverse transcription

## Abstract

Transfer RNAs undergo diverse posttranscriptional modifications to regulate a myriad of cellular events including translation, stress response, and viral replication. These posttranscriptional modifications are synthesized by site-specific modification enzymes. Recent RNA-seq techniques have revealed multiple features of tRNA such as tRNA abundance, tRNA modification, and tRNA structure. Here, we adapt a tRNA-sequencing technique and design a new functional analysis where we perform mutational profiling of tRNA modifications to gain mechanistic insights into how tRNA modification enzymes recognize substrate tRNA. Profiling of *Geobacillus stearothermophilus* tRNAs and protein orthology analysis predict the existence of natural modifications in 44 tRNA molecular species of *G. stearothermophilus*. We selected the 1-methyladenosine modification at position 22 (m^1^A22) and tRNA (m^1^A22) methyltransferase (TrmK) for further analysis. Relative quantification of m^1^A22 levels in 59 tRNA transcripts by mutational profiling reveals that TrmK selectively methylates a subset of tRNAs. Using 240 variants of tRNA^Leu^ transcripts, we demonstrate the conserved nucleosides including U8, A14, G15, G18, G19, U55, Purine57, and A58 are important for the methyl transfer reaction of TrmK. Additional biochemical experiments reveal that TrmK strictly recognizes U8, A14, G18, and U55 in tRNA. Furthermore, these findings from tRNA^Leu^ variants were crossvalidated using variants of three different tRNA species. Finally, a model of the TrmK–tRNA complex structure was constructed based on our findings and previous biochemical and structural studies by others. Collectively, our study expands functional analyses of tRNA modification enzyme in a high-throughput manner where our assay rapidly identifies substrates from a large pool of tRNAs.

Transfer RNAs are essential adapter molecules that decode the genetic information in mRNAs during protein synthesis. Cellular tRNAs undergo a large level of posttranscriptional modification with nearly 170 known modifications across the three domains of life (1). Transfer RNA modifications contribute to stabilization of tRNA structure, codon–anticodon interactions, prevention of frame-shift errors, and recognition by aminoacyl-tRNA synthetases and translation factors (2, 3, 4, 5, 6, 7). Therefore, tRNA modifications play diverse roles in translation. In addition, rates of tRNA modification vary in response to environmental stresses such as heat stress, starvation, oxidative stress, and antibiotic stress (8, 9, 10, 11, 12). In the case of eukaryotes, tRNA modifications affect tRNA splicing, processing, transport of tRNA, quality-control systems of tRNA, and RNA interference and regulate higher biological phenomena such as oncogenesis, infection, and immune response (12, 13, 14, 15, 16, 17, 18, 19, 20). In fact, malfunctions of tRNA modification cause genetic diseases (20, 21, 22). The majority of tRNA modifications are synthesized by site-specific tRNA modification enzymes. It is, thus, critical to identify the enzymes responsible for tRNA modification and determine the chemical structure, location, and biological function of tRNA modifications.

To accomplish this, various hierarchical approaches have been established in the past 2 decades. For example, comparative genomics can be utilized to select candidate genes required for tRNA modifications of interest (23, 24, 25, 26, 27). To validate the candidates, reverse genetics and biochemical analyses are often used. More specifically, to see if the gene product can synthesize the target modification, the nucleosides in a single tRNA species are profiled using mass spectrometry or classical biochemical techniques such as Kuchino’s postlabeling method (28, 29, 30, 31). With this strategy, the identification of whole sets of *Escherichia coli* tRNA modifications and the enzymes responsible has recently been completed (32). This approach, however, requires purification of each tRNA, which can be a major effort. Furthermore, many organisms are not genetically manipulatable.

To tackle the obstacle, high-throughput tRNA-sequencing techniques have been developed for comprehensive tRNA analyses (*e.g.* tRNA Modomics, tRNA abundance, and tRNA structurome) (33, 34, 35, 36, 37, 38, 39, 40). These techniques rely on a common basic strategy wherein tRNA sequences are converted into complementary DNA (cDNA) by reverse transcription (RT). During RT, some RNA modifications induce termination and/or nucleotide misincorporation into the cDNA which are recorded as RT stop counts and elevated mutation rates, respectively, in next-generation sequencing (40). These RT-derived signals allow one to predict the modification sites in tRNA. For example, Kimura et al. predicted new tRNA modifications in *Vibrio cholerae* tRNAs from the RT signals and identified them using RNA mass spectrometry (37). Alternatively, Marchand et al. developed AlkAniline-Seq wherein 7-methylguanosine (m^7^G)-, 3-methylcytidine (m^3^C)-, and dihydrouridine (D)-specific chemical cleavages by aniline are utilized. Thus, AlkAniline-Seq is a powerful tool for profiling m^7^G, m^3^C, and D positions in RNAs at single-nucleotide resolution (36). Very recently, Behrens et al. developed mim-tRNAseq where the optimized experimental procedure and computation for tRNA sequencing data provide accurate information about tRNA abundance and modification status in any organism (39). Moreover, Yamagami et al. developed tRNA Structure-seq that predicts cellular tRNA secondary structures wherein both mutations in cDNA from natural tRNA modifications and chemical modifications from dimethyl sulfate that probes RNA secondary structure are simultaneously detected by Mutational Profiling (MaP) (40). However, despite the improvement of these tRNA sequencing techniques, to date none have been applied to functional analyses of tRNA modification enzyme.

In this report, we first describe a new application of a tRNA-sequencing technique that provides keen insight into the enzymatic properties of a tRNA modification enzyme. We employ the tRNA Structure-seq methodology with minor protocol changes (here, we call it tRNA-MaP; see Experimental procedures) and sequence native tRNAs from *Geobacillus stearothermophilus* which is a thermophilic eubacterium with an optimal growth temperature of 55 ∼ 60 °C (41). While modified nucleosides in tRNA mixture from *G. stearothermophilu*s was recently analyzed by LC-MS (42), only four tRNA sequences have been reported (43, 44, 45, 46, 47). In this study, our protein orthology analysis proposes that at least 40 tRNA modification enzymes are conserved in *G. stearothermophilus*. Of these ortholog proteins, we focus on tRNA (m^1^A22) methyltransferase (TrmK) that methylates the 1-nitrogen atom of adenosine at position 22 in tRNA for the following reasons. First, the *trmK* gene is widely found in Gram-positive bacteria and some Gram-negative bacteria, and a homolog of *trmK* is also encoded in the *G. stearothermophilus* genome (25). Second, m^1^A gives a clear RT signal as the methylation occurs on the Watson-Crick face. Finally, a crystal structure (PDB: 6Q56) and extensive biochemical data for *Bacillus subtilis* TrmK are available (48), which provides a good benchmark. Herein, we study the enzymatic properties of TrmK *via* rapid screening of 240 tRNA variants using tRNA-MaP and further examine the detailed mechanism of the substrate recognition using already-established methods. We report the advantages of tRNA-MaP technology in combination with the already-established methods in the enzymatic analysis of tRNA modification enzymes.

## Results

### tRNA-MaP detects multiple RT signals from natural tRNA modifications

**Figure 1:**
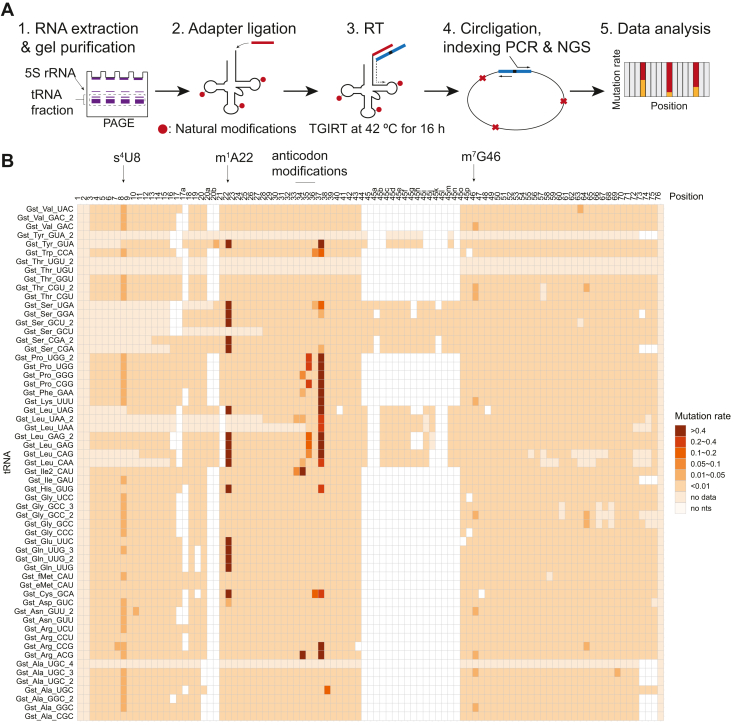
**tRNA-MaP detects various natural tRNA modifications.***A*, workflow for tRNA-MaP is illustrated. Detailed procedures are provided in the Experimental procedures section. *B*, mutation rates from natural modifications in *Geobacillus stearothermophilus* tRNAs upon analysis by tRNA-MaP are provided. MaP, Mutational Profiling.

To see which modified nucleosides give the strong RT-signals in *G. stearothermophilus* tRNA, we adapted the tRNA Structure-seq methodology with a minor protocol change, wherein RT-derived signals from natural modifications are recoded as mutations in cDNA (Fig. 1*A*). In the previous study, tRNA Structure-seq detected about 40% of *E. coli* natural tRNA modifications including 4-thiouridine (s^4^U), D, pseudouridine (Ψ), 2′-*O*-methylated nucleosides, various anticodon modifications, m^7^G, aminocarboxypropyluridine (acp^3^U), and 5-methyluridine (m^5^U) (40). In the original protocol, the RT reaction is performed using Marathon RTase in the presence of MnCl_2_. We changed the enzyme to TGIRT as an optimized protocol for the RT of tRNA is available (39). Since each replicate showed similar mutation rates (Fig. S1), we combined the replicates and analyzed the combined data. We used 59 reference tRNA genes encoded in the *G. stearothermophilus* genome for the MaP analysis. From 44 tRNAs, more than 1000 read counts were obtained and nearly all nucleotide positions could be detected. However, 15 tRNAs were not able to be analyzed by the MaP. Enough read counts for the MaP analysis were not obtained from four tRNAs (tRNA^Ala_UGC_4^, tRNA^Thr_UGU^, tRNA^Thr_UGU_2^, and tRNA^Tyr_GUA_2^), suggesting that these tRNAs are poorly expressed (or not expressed) in the cells under the culture condition. The other 11 tRNAs (tRNA^Leu_CAA, CAG, UAA, UAA_2, UAG^, tRNA^Ser_CGA, CGA_2, GCU, GCU_2, GGA, UGA^) lacked some portions of the data. In the cases of these tRNAs, the RT reaction was likely to be stalled due to their low expression levels, modified nucleosides, and/or rigid local structures in these tRNAs. Nonetheless, tRNA-MaP detected various RT-derived signals from natural modifications in the native tRNAs (Fig. 1*B*). We also conducted a protein orthology analysis to search for tRNA modification enzymes that are conserved in *G. stearothermophilus*. We retrieved tRNA modification enzymes previously identified in *B. subtilis* (27) and performed a Blast search, which identified homologs of 40 tRNA modification enzymes conserved in *G. stearothermophilus* (Table S1). With the combined results from tRNA-MaP and the protein orthology analysis, we predicted provisional tRNA modification enzymes and modifications in *G. stearothermophilus* (Fig. S2). For example, 32 out of 44 tRNAs gave high mutation rates (defined as a mutation rate > 0.01) at position 8. Of the tRNA modification enzyme genes encoded in *G. stearothermophilus*, tRNA s^4^U synthetase (ThiI) homolog would catalyze s^4^U8 formation since ThiI is the only tRNA modification enzyme, which acts on U8 (Fig. 1*B*). Similarly, 18 tRNAs had notably high mutation rates at position 22. TrmK homolog would catalyze the m^1^A22 formation since TrmK is the only tRNA modification enzyme, which acts on A22 (Fig. 1*B*). Our analysis shows that TrmK homolog methylates A22 in tRNA^Asp^, tRNA^Cys^, tRNA^Gln^, tRNA^Glu^, tRNA^His^, tRNA^Leu^, tRNA^Ser^, and tRNA^Tyr^ (Fig. 1*B*). It should be mentioned that the recombinant *B. subtilis* TrmK methylates the same subset of tRNA transcripts *in vitro* (48). Furthermore, although enough read counts were not obtained from three tRNAs (tRNA^Ser_GCU^, tRNA^Leu_UAA, UAA_2^), these tRNAs probably contain the m^1^A22 modification because these tRNA transcripts were methylated by TrmK. Thus, our experimental results suggest that *G. stearothermophilus* TrmK methylates the 21 tRNA molecular species. We also detected multiple sites that possess high mutation rates in the anticodon loop (at positions 34, 35, and 37). Given that *G. stearothermophilus* has a similar gene set of tRNA modification enzymes responsible for anticodon modifications as *B. subtilis* (27), we can speculate about tentative tRNA modifications and the responsible modification enzymes. For example, we predict that TadA converts A34 to inosine 34 in tRNA^Arg^; TilS catalyzes lysidine 34 formation in tRNA^Ile2^; TrmL methylates C34 in tRNA^Leu_CAA^. Overall, tRNA-MaP and protein orthology analysis enabled us to predict modifications conserved in native tRNAs in *G. stearothermophilus*.

### Characterization of methyl-transfer activity of recombinant *G. stearothermophilus* TrmK

**Figure 2:**
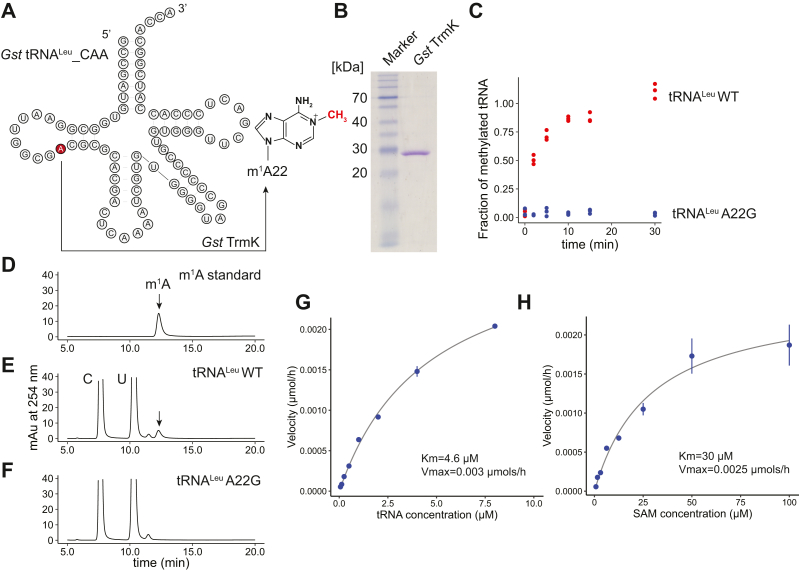
***Geobacillus stearothermophilus* TrmK catalyzes the formation of m**^**1**^**A at position 22 in tRNA.***A*, TrmK methylates A22 at the end of the D-loop using S-adenosyl-L-methionine (AdoMet) as a methyl group donor. *B*, a 10% SDS-PAGE analysis shows high purity of the recombinant TrmK. The SDS-PAGE gel was stained with Coomassie Brilliant Blue. *C*, *G. stearothermophilus* TrmK activity was measured with tRNA^Leu_CAA^ WT and A22G variants and ^3^H-AdoMet at specific time points (0, 2, 5, 10, 15, and 30 min) where 100 nM TrmK was used. The experiments were independently performed in triplicate (n = 3). *D*–*F*, tRNAs methylated by TrmK were subjected to nucleoside analysis. HPLC chromatograms of (*D*) m^1^A standard, (*E*) tRNA^Leu^ WT methylated by TrmK, and (*F*) tRNA^Leu^ A22G methylated by TrmK are provided. *G* and *H*, kinetic analyses of TrmK for (*G*) tRNA^Leu^ and (*H*) AdoMet. The TrmK reaction was performed at 60 °C for 2 min in the presence of 100 nM TrmK and (*G*) 50 μM AdoMet and (*H*) 5 μM tRNA, respectively. The apparent K_m_ and V_max_ were obtained from the fitting parameters of the Michaelis–Menten equation. The experiments were independently performed in triplicate (n = 3).

Given that some tRNA modifications leave strong footprints during RT, tRNA-MaP should be applicable to functional analyses of tRNA modification enzyme. To test this idea, we selected TrmK as a model enzyme. TrmK methylates the 1-nitrogen atom of adenine at position 22 in tRNA using S-adenosyl-L-methionine (AdoMet) as a methyl group donor resulting in the formation of m^1^A22 (Fig. 2*A*). Since the methylation occurs on the Watson-Crick face, m^1^A gives a strong RT signal. Indeed, we observed remarkable mutation rates at position 22 in some native tRNAs (Fig. 1*B*). To perform functional analyses on TrmK, we first purified recombinant *G. stearothermophilus* TrmK (Fig. 2*B*) and tested its methyltransferase activity (Fig. 2*C*). TrmK shows catalytic activity when the tRNA^Leu_CAA^ WT transcript is used as a substrate. To determine whether the methylation site was A22, we prepared a tRNA^Leu_CAA^ mutant in which A22 was substituted with G (tRNA^Leu_CAA^ A22G). The tRNA^Leu_CAA^ A22G transcript was not methylated by TrmK (Fig. 2*C*). Furthermore, nucleoside analysis produced m^1^A in the tRNA^Leu^ WT transcript, but not in the tRNA^Leu^ A22G transcript (Fig. 2, *D*–*F*). These results demonstrate that *G. stearothermophilus* TrmK catalyzes m^1^A22 formation in tRNA. We also performed a kinetic analysis of TrmK in which the kinetic parameters K_m_ and V_max_ for tRNA^Leu_CAA^ transcript were determined to be 4.6 μM and 0.003 μmols/h, respectively (Fig. 2*G*). The Km value was one order of magnitude greater than that for other tRNA methyltransferases such as *Thermus thermophilus* TrmI and TrmH, while the V_max_ value is comparable to those enzymes (49, 50). The kinetic parameters K_m_ and V_max_ for AdoMet were also determined and shown to be 30 μM and 0.0025 μmols/h, respectively (Fig. 2*H*).

### tRNA-MaP detects the m^1^A22 modification in 20 tRNA transcript species of the 59 tRNA transcript species tested

**Figure 3:**
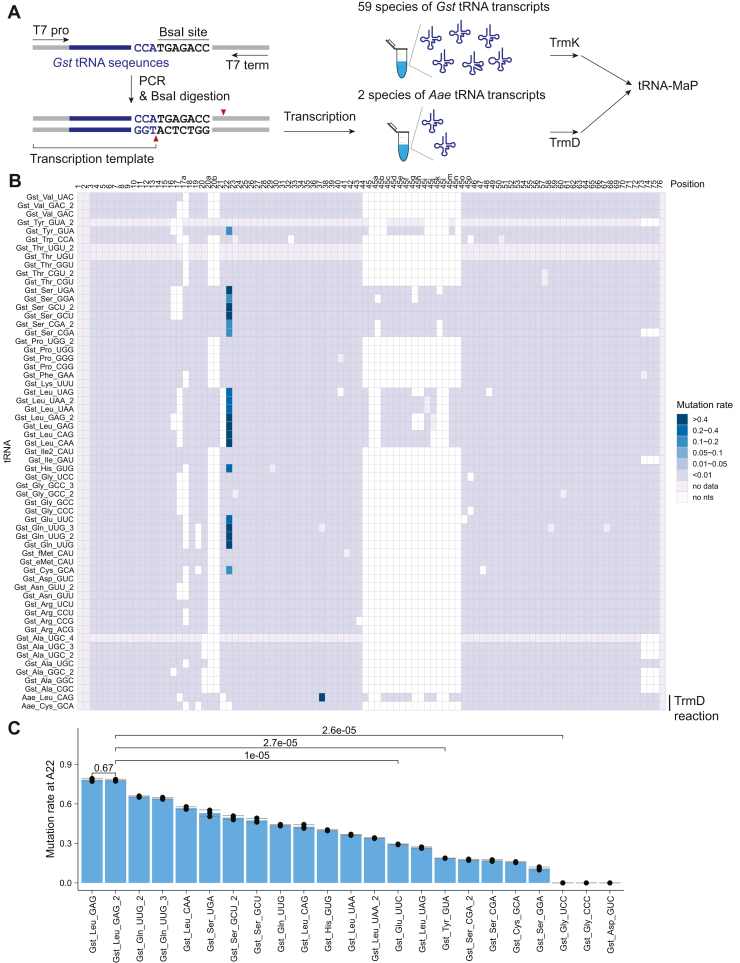
**tRNA-MaP successfully detects m**^**1**^**A sites in tRNA transcripts methylated by recombinant TrmK *in vitro*.***A*, workflow of *in vitro* T7 transcription to prepare a mixture of 59 species of *Geobacillus strearothermophilus* tRNA transcripts. *Aquifex aeolicus* tRNAs were also prepared separately. The detailed protocol is provided in the Experimental procedures section. In brief, the tRNA-MaP experiment was performed in triplicates (n = 3). The TrmK reaction was conducted in the presence of 100 nM TrmK, 2 μM tRNA mixture, and 10 μM AdoMet at 60 °C for 10 min, whereas the TrmD reaction was performed in the presence of 400 nM TrmD, 2 μM tRNA mixture, and 10 μM AdoMet at 55 °C for 5 min. *B*, mutation rates from *G. stearothermophilus* tRNAs (methylated with TrmK) and *A. aeolicus* tRNAs (methylated with TrmD) upon analysis by tRNA-MaP are provided. *C*, the mutation rates at A22 in 23 tRNAs are provided. Errors are SD from three biological replicates (n = 3). MaP, Mutational Profiling.

**Figure 4:**
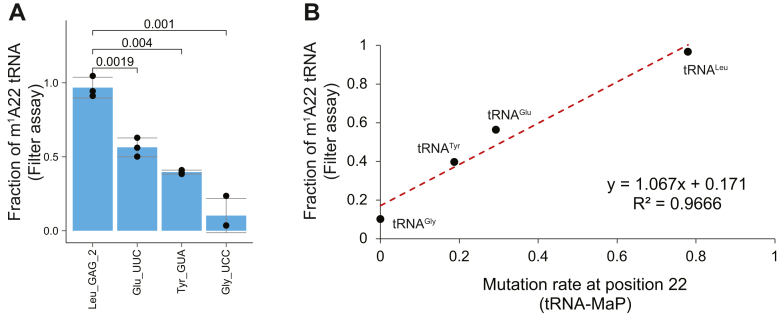
**tRNA-MaP results are reproduced by a classical filter assay.***A*, the methyl group acceptance activities of tRNA^Leu_GAG_2^, tRNA^Glu_UUC^, tRNA^Tyr_GUA^, and tRNA^Gly_UCC^ were measured by filter assay. The experiments were independently performed in triplicate (n = 3). *B*, the mutation rates at position 22 in tRNA^Leu_GAG_2^, tRNA^Glu_UUC^, tRNA^Tyr_GUA^, and tRNA^Gly_UCC^ from tRNA-MaP are compared with the methyl group acceptance activity from the filter assay. The values from three independent experiments are averaged and plotted. MaP, Mutational Profiling.

To prepare a mixture of *G. stearothermophilus* tRNA transcripts, we designed DNA oligonucleotides that encode the BsaI restriction site downstream of the 3′ end of the tRNA gene (Fig. 3*A*). The BsaI digestion generates DNA templates ending with GGT-5’ (which is converted into the CCA terminus during transcription). In this way, we were able to prepare 59 species of *G. stearothermophilus* tRNA transcripts in a single transcription reaction. These tRNAs were treated with TrmK and subjected to tRNA-MaP (Fig. 3*A*). To benchmark our method, we prepared two *Aquifex aeolicus* tRNA transcripts (tRNA^Leu^ and tRNA^Cys^) and tRNA (m^1^G37) methyltransferase TrmD. The substrate tRNA recognition by TrmD is well-studied (51) and of tRNA^Leu^ and tRNA^Cys^, TrmD only methylates tRNA^Leu^ (51). In addition, m^1^G inhibits Watson-Crick base pairing and thus provides a strong RT signal. To assess whether tRNA-MaP detects the m^1^G37 modification, we spiked two *A. aeolicus* tRNA transcripts, which were treated with TrmD, into the *G. stearothermophilus* tRNA mixture and conducted the tRNA-MaP experiment (Fig. 3*A*). We calculated the mutation rates in each transcript (Figs. 3*B* and S3) and detected a strong mutation signal at position 37 of TrmD-reacted *A. aeolicus* tRNA^Leu^, but not in tRNA^Cys^ (Figs. 3*B*, S3, *A* and *B*). Based on the mutation rates at position 22, we detected that *G. strearothermophilus* TrmK methylates 20 tRNA transcripts with the exception of tRNA^Asp_GUC^ which was detected to be methylated in native tRNAs (Compare Figs. 1*B* and 3*B*). These results indicate that tRNA-MaP can be used to determine the tRNA specificity of modification enzymes. We also found that the A22 mutation rates vary depending on the tRNA species (Fig. 3*C*). For example, tRNA^Leu_GAG^ and tRNA^Leu_GAG_2^ showed the highest mutation rate of ∼0.79 across all transcripts, whereas tRNA^Glu_UUC^ showed a statistically significantly lower mutation rate than tRNA^Leu_GAG_2^ (Fig. 3*C*). In addition, tRNA^Tyr_GUA^ and tRNA^Gly_UCC^ show even lower mutation rates with *p*-values of 2.7e-05 and 2.6e-05, respectively (Fig. 3*C*). To test if the difference of the mutation rates between these tRNAs were derived from m^1^A22 levels, we performed a classical filter assay where we monitored the ^3^H-methyl group acceptance activities of the four tRNA transcripts (tRNA^Leu^, tRNA^Glu^, tRNA^Tyr^, and tRNA^Gly^) (Fig. 4*A*). The fraction of methylated tRNA^Leu_GAG_2^ was indeed exceptionally high (∼1.0), whereas the fraction of methylated tRNAs from the other three species were significantly lower than that of tRNA^Leu_GAG_2^ with *p*-values of less than 0.005 (Fig. 4*A*). The correlation plot between the mutation rates from tRNA-MaP and the fraction of methylated tRNA from the filter assays were well-fitted to a linear regression line with an r^2^ value of 0.9666 (Fig. 4*B*), demonstrating that tRNA-MaP is quantitatively comparable to the ^3^H-methyl group tracing filter assay. We observed a relatively weak mutation signal in the native tRNA^Asp^ (Fig. 1*B*), but not in the tRNA^Asp^ transcript (Figs. 3*B* and S4*A*). Gel-based assays reproduced that the tRNA^Asp^ transcript does not show the methyl group acceptance activity under the tested condition (Fig. S4, *A* and *B*). This inconsistency between native tRNA^Asp^ and transcript may be explainable by the difference in reaction conditions such as different tRNA populations, different reaction times, and presence/absence of molecular crowding. Particularly, the tRNA population in the synthetic pool and reaction time might largely affect the substrate specificity in the steady-state condition: the methyl-transfer reaction was stopped before the transcripts were fully modified. Indeed, the estimated tRNA abundance from the read counts in the synthetic tRNA transcript mixture is quite different from natural tRNAs where the relative abundance of tRNA^Asp^ transcript is estimated to be ∼32-fold lower than the native tRNA^Asp^ (Fig. S5). Overall, tRNA-MaP results suggest that TrmK has a selective mechanism (*i.e.*, tRNA preference) for its substrate tRNAs.

### Application of tRNA-MaP for determination of the substrate tRNA recognition mechanism of TrmK

To study how a tRNA methyltransferase recognizes a substrate tRNA, a series of tRNA variants that have a point mutation at a specific position can be examined by incorporation of methyl group to determine how the point mutation alters the methyl group acceptance activity in the tRNA (10, 48, 52, 53, 54, 55, 56). For example, if a point mutation in a given tRNA diminishes methyl group acceptance activity, then the mutation site is directly recognized or indirectly sensed *via* alternation of tRNA structure by that tRNA methyltransferase. While the measurement of radioisotope-labeled methyl group incorporation into tRNA transcript by filter assay is useful for such functional analysis, it is low-throughput and is therefore not suitable for dealing with a large number of tRNA variants. To address this issue in a high-throughput manner, we applied tRNA-MaP. We designed a series of tRNA^Leu_GAG_2^ variants with point mutations at all bases at 80 different positions, for a total of 240 tRNA^Leu_GAG_2^ variants (80 positions × 3 mutations/position; see Table S2). ShapeMapper 2 executes an alignment-based variant-calling method to count the mutations in each read. Given the alignment-based method, it is not suitable for sequence reads with an extremely low sequence diversity like the single-point variant library. To avoid ambiguous calculations of mutation rates, herein, we took a different strategy using Seqkit (57). We extracted a specified sequence from fastq files to directly count the read number of the specified sequence. In the Seqkit analysis, we used four specified sequences that have either A22, U22, C22, or G22 and counted the read number for each variant (see Experimental procedures). This strategy avoids sequence alignment and instead counts the actual number of reads of a specified sequence. This method, thus, can be applied to sequences that have an extremely low diversity. To verify this strategy, we compared the mutation rates at A22 calculated from ShapeMapper 2 and those from the Seqkit analysis with *G. stearothermophilus* native tRNAs (Fig. S6). We found that both mutation rates from ShapeMapper and the Seqkit analysis have a good correlation with an r^2^ value of 0.99 (Fig. S6).

**Figure 5:**
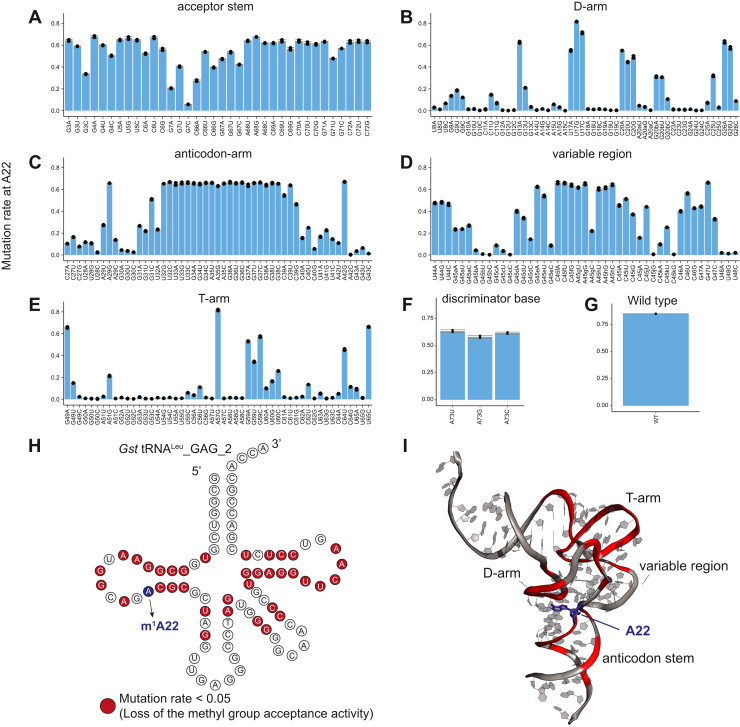
**tRNA-MaP detects the methyl group acceptance activities in 240 different tRNA variants.***A*–*F*, the methyl group acceptance activities (mutation rates at A22) of the tRNA^Leu_GAG_2^ variants in (*A*) the acceptor stem, (*B*) D-arm, (*C*) anticodon-arm, (*D*) variable region, (*E*) T-arm, and (*F*) discriminator base upon analysis by Seqkit are provided. The experiments were independently repeated in duplicate (n =2). *G*, the methyl group acceptance activity of the tRNA^Leu_GAG_2^ WT is provided. The experiments were independently repeated in triplicate (n =3). *H* and *I*, the nucleotides where methyl group acceptance activities (mutation rates at A22) less than 0.05 (highlighted by *red*) in the tRNA^Leu^ variants are mapped onto (*H*) the secondary structure and (*I*) the tertiary structure. The tertiary structure of tRNA^Leu^ was retrieved from the structure of the complex of *Thermus thermophilus* Leucyl-tRNA synthetase with tRNA^Leu^ transcript (PDB: 2BYT). MaP, Mutational Profiling.

**Figure 6:**
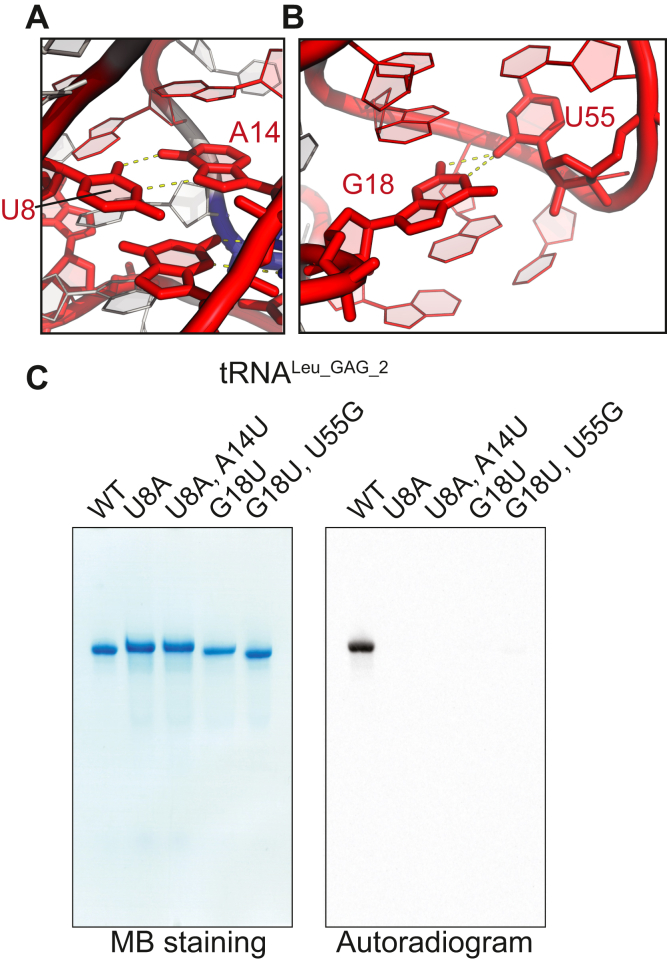
**TrmK recognizes the U8, A14, G18, and U55 in tRNA**^**Leu**^**.***a* and *b*, representative tertiary interactions of (*A*) U8-A14 and (*B*) G18-U55 from the complex of *Thermus thermophilus* Leucyl-tRNA synthetase with tRNA^Leu^ transcript (PDB: 2BYT) are illustrated. The nucleotides in the tRNA^Leu^ variants where methyl group acceptance activities (mutation rates at A22) less than 0.05 are highlighted in *red*. *C*, the methyl group acceptance activities in tRNA^Leu_GAG_2^ WT, U8A, U8A-A14U, G18U, and G18U-U55G were measured with a gel assay. The TrmK reaction was performed in the reaction mixture containing 50 mM Tris–HCl (pH 7.6), 200 mM KCl, 5 mM MgCl_2_, 0.0125 OD/μl tRNA, 50 μM ^14^C-S-adenosyl-L-methionine (AdoMet), and 100 mM TrmK at 60 °C for 10 min. The gel was stained with Methylene blue (MB), and ^14^C-methyl group incorporation was monitored by autoradiography.

We then prepared a mixture of 240 tRNA^Leu^ variants by *in vitro* transcription and performed tRNA-MaP (Fig. 5). The tRNA variants in the acceptor stem retained high mutation rates at A22 (= high methyl group acceptance activity) with the exception of some variants such as G7C and G7A, suggesting that the acceptor stem is not strictly recognized by TrmK (Fig. 5*A*). Variations in U8∼A15, G18∼G19, A20a, and C23∼C25 in the D-arm drastically decreased the mutation rates at A22 (<0.05 mutation rates) (Fig. 5*B*). The variants in which G13 was substituted with pyrimidine bases significantly decreased the mutation rates, which is consistent with the previous report by Dégut *et al.* (48). G18 and G19 in the D-loop and U55 and C56 in the T-loop form a three-dimensional interaction. This interaction is crucial for the tRNA recognition as the G18 and G19 variants completely lost their methyl group acceptance activities (Fig. 5*B*). Furthermore, the D-stem structure must be formed for TrmK to recognize tRNA as a substrate (Fig. 5*B*). Moreover, the anticodon stem is also recognized by TrmK but the anticodon loop is not (Fig. 5*C*). In the variable region, variations in G45b, G45c, C45j, and C45k led to loss of methyl group acceptance activity, implying that the stem structure of the variable region in tRNA^Leu^ is also important for tRNA recognition (Fig. 5*D*). G45b, G45c, C45j, and C45k correspond to G_e11_, G_e12_, G_e2_, and G_e3_ in the reference (58). In addition to the D-arm region, the T-arm structure is required for recognition by TrmK (Fig. 5*E*). For example, variations in U55 and C56 resulted in a significant drop in the mutation rates due to the loss of the tertiary interaction with the D-loop (Fig. 5*E*). U54 and A58 form a reverse Hoogsteen base pair which is integral to the T-loop fold (59) and variations here also diminished methyl group acceptance activity (Fig. 5*E*). A point mutation in the T-stem also decreased the activity (Fig. 5*E*). Finally, the discriminator base does not appear to affect tRNA recognition by TrmK (Fig. 5*F*). We mapped the mutated positions that resulted in significantly decreased mutation rates at A22 onto the structure of the tRNA (Fig. 5, *H* and *I*). The conserved nucleotides such as U8, A14, G18, G19, U54, U55, C56, and A58 are involved in the formation of the L-shaped structure (60). Because variations at these nucleotides significantly decreased the methyl group acceptance activity (Fig. 5*H*), we propose that TrmK recognizes the three-dimensional core of tRNA^Leu^. In addition to the tertiary contacts, we observed that disruption of a base pair in the anticodon stem decreases the mutation rate (Fig. 5*H*), while the A29G and A42G variants retain similar methyl group acceptance activities to WT. The A29G and A42G variants can form G29-U41 and U28-G42 base pairs in the stem, respectively. Therefore, the anticodon-stem structure itself is important for the methyl group acceptance activity. Thus, these results suggest that the phosphate-ribose backbone of the anticodon stem is recognized by TrmK. To verify whether the U8-A14 and G18-U55 tertiary base pairs is important for the methylation by TrmK, we individually prepared tRNA^Leu_GAG_2^ variants, in which U8–A14 or G18–U55 interaction was disrupted and performed gel assays (Fig. 6, *A* and *B*). The U8A and G18U variants completely lost the methyl group acceptance activity, reproducing the tRNA-MaP result (Fig. 6*C*). To our surprise, the compensatory mutations of U8A-A14U and G18U-U55G did not rescue the methyl group acceptance activity (Fig. 6*C*). These results suggest that U8, A14, G18, and U55 nucleosides themselves are recognized by TrmK, although there is a possibility that the compensatory mutations alter the local structure of tRNA by steric hindrance and/or formation of unexpected tertiary interaction.

**Figure 7:**
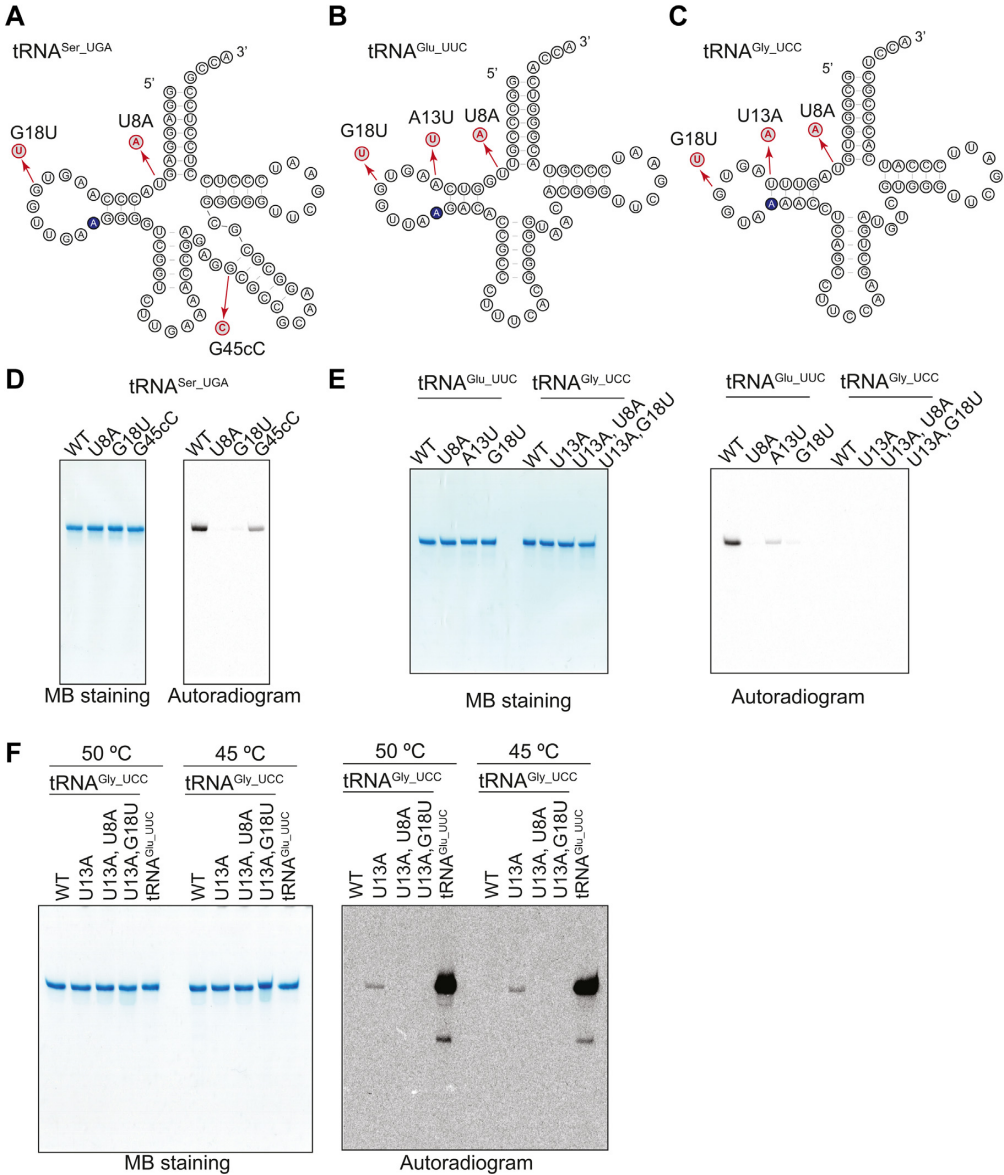
**TrmK recognizes the U8 and G18 in other tRNA species.***A*–*C*, secondary structures of (*A*) tRNA^Ser_UGA^, (*B*) tRNA^Glu_UCC^, and (*C*) tRNA^Gly_UCC^ are drawn. *D*, the methyl group acceptance activities in tRNA^Ser_UGA^ WT, U8A, G18U, and G45cC were measured by a gel assay. The reaction was performed at 60 °C for 10 min. *E*, the methyl group acceptance activities in WT, U8A, A13U, and G18U for tRNA^Glu_UUC^ and WT, U13A, U13A_U8A, and U13A_G18U for tRNA^Gly_UCC^ were measured. The reaction was performed at 60 °C for 10 min. *F*, the methyl group acceptance activity in the tRNA^Gly_UCC^s was measured. The reaction was performed at 50 °C or 45 °C for 30 min. The contrast of the gel image was adjusted due to the slight methyl group acceptance activity in tRNA^Gly^ as compared with that in tRNA^Glu^.

Some tRNA modification enzymes are known to selectively install modifications on their substrate tRNAs with different tRNA recognition modes. For example, In *Saccharomyces cerevisiae*, Trm140 catalyzes the m^3^C32 formation (39, 61, 62). Trm140 has two distinct tRNA recognition modes: one is that Trm140 recognizes the G35U36 and *N*^6^-threonylcarbamoyl adenosine (t^6^A37) in tRNA^Thr^ as key elements. The other is that Trm140 recognizes the i^6^A37 in tRNA^Ser^ bound with the seryl-tRNA synthetase (61). We considered that TrmK may possess multiple tRNA recognition modes like Trm140. To test this idea, we performed biochemical assays using tRNA^Ser_UGA^, tRNA^Glu_UUC^, and tRNA^Gly_UCC^ transcripts with relevant point mutations (Fig. 7, *A*–*C*). We observed that the U8 and G18 residues in the tRNA^Ser^ and tRNA^Glu^ transcripts are also important like those in the tRNA^Leu^ transcript (Fig. 7, *D* and *E*). Likewise, the variations of G45cC in tRNA^Ser^ and A13U in tRNA^Glu^ clearly decrease the methyl group acceptance activity to ∼20% as compared with WT (Fig. 7, *D* and *E*). Thus, the crucial residues on the tRNA recognition by TrmK are conserved in the substrate tRNAs tested. In the case of *B. subtilis*, tRNA^Gly^ possesses a U13-A22 Watson-Crick base pair. It has been reported that this Watson-Crick base pair acts negatively on the methyl-transfer activity of *B. subtilis* TrmK (48). Because the sequence of *G. stearothermophilus* tRNA^Gly^ is completely identical to that of *B. subtilis* tRNA^Gly^, we considered that the U13-A22 base pair in *G. stearothermophilus* tRNA^Gly^ might act negatively on the methylation by *G. stearothermophilus* TrmK. To confirm this idea, we prepared the tRNA^Gly^ U13A transcript (Fig. 7*C*). Unexpectedly, this variant was not methylated by TrmK at 60 °C (Fig. 7*E*). The L-shaped structure of the tRNA^Gly^ U13A variant is thought to be relatively unstable at 60 °C since the D-stem in this variant is composed of two U-A and one G-C base pairs only (Fig. 7*C*). Therefore, we performed the assay at 50 °C and 45 °C and could detect slow methylation of tRNA^Gly^ U13A transcript. We further designed double mutations of U8A or G18U in tRNA^Gly^ U13A (Fig. 7*C*). These double mutants were not methylated by TrmK, indicating that the U8 and G18 in tRNA^Gly^ are also required for the methylation by TrmK (Fig. 7, *E* and *F*). Altogether, we conclude that the U8 and G18 in tRNA^Ser^, tRNA^Glu^, and tRNA^Gly^ are the important residues for the substrate recognition by TrmK. The tRNA^Gly^ WT is not methylated by TrmK due to the U13-A22 base pair, and this enzymatic property of *G. stearothermophilus* TrmK is the same as that of *B. subtilis* TrmK. Similar to tRNA^Leu^, stabilization of the stem structure of the variable region in tRNA^Ser^ accelerates the TrmK reaction.

**Figure 8:**
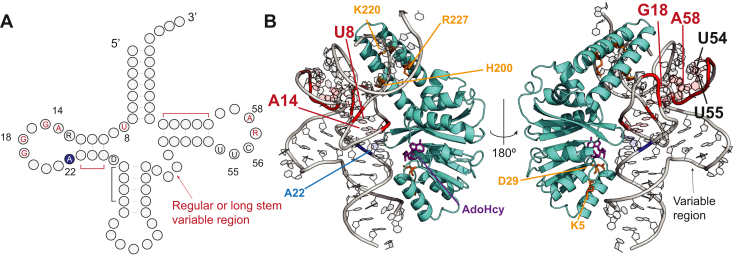
**Structure modeling with the experimental restraints provides a reasonable structure of the TrmK–tRNA complex.***A*, the tRNA recognition mechanism of TrmK. The nucleotides and stem structure (*lines*) shown in the secondary structure are recognized by TrmK. The *red* colored are the critical regions of the tRNA recognition mechanism identified in this study using tRNA-MaP. The nucleotide symbols R and D are purine (G or A) and the nucleotides (G or A or U), respectively. *B*, the structure of the TrmK–tRNA^Leu^ complex was modeled by HDOCK with the input of experimental restraints (see the Experimental procedures section) (63). Amino acid residues of TrmK key for methylation are highlighted in *yellow*. The color code is the same with (*A*). The TrmK structure (PDB: 6Q56) and tRNA^Leu^ structure (PDB: 2BYT) were used. The *Mycoplasma capricolum* TrmK structure (transparent) complexed with AdoHcy (*purple*) was superposed onto the model. MaP, Mutational Profiling.

We summarized the tRNA recognition mechanism of TrmK (Fig. 8*A*). TrmK requires the structure elements of D-stem, T-stem, and a portion of anticodon-stem (Fig. 8*A*). Albeit the substrate recognition by TrmK was previously reported (48), we add new knowledge on the recognition mechanism which is experimentally proofed in this study. For example, the tRNA-MaP experiment suggested that U8, A14, G15, G18, G19, U54, U55, Purine57, and A58 nucleotides are important for the TrmK reaction. Then, our biochemical assays suggested that U8, A14, G18, and U55 bases themselves are recognized by TrmK. Also, TrmK prefers a compacted variable region (*i.e.*, regular or long variable region with a stabilized stem structure) (Fig. 8*A*). Finally, we performed structure modeling of the TrmK–tRNA complex using the tRNA-MaP data and key TrmK amino acid residues (48) as experimental restraints (Fig. 8*B*). We used the crystal structures of *B. subtilis* TrmK (PDB: 6Q56) and *T. thermophilus* tRNA^Leu^ (PDB:2BYT) for the modeling on HDOCK software (63). We, then, superimposed the AdoHcy-binding site of *Mycoplasma capricolum* TrmK structure (PDB: 6QE6) on our complex model to evaluate distance and orientation between the target residue A22 in tRNA and the catalytic pocket in TrmK. Our TrmK–tRNA complex model suggests that TrmK recognizes nearly the entire L-shape structure except for the anticodon loop, CCA terminus, and the acceptor stem (Fig. 8*B*). Furthermore, the U8-A14 and G18-U55 tertiary contacts are orientated to TrmK. More importantly, the A14 and G18 residues are spatially close to the TrmK surface (Fig. 8*B*). On the other hand, the reverse Hoogsteen base pair between U54 and A58 is at the opposite side of the TrmK-binding region. These results imply that the tertiary contacts probably contribute to adjust the angles and distances between U8, A14, G18, U55, and the methylation site A22. Moreover, our model suggests that although the variable region has no contact with TrmK, the structural compactness in the variable region by stabilization of the stem structure probably reduces steric hindrance between tRNA and TrmK. The Watson-Crick face of the A22 base is orientated directly toward the S-adenosyl-L-homocysteine (AdoHcy)-binding site, although the N1 atom is ∼7.6 Å away from the AdoHcy-binding site (Figs. 8*B* and S7 see Discussion). Therefore, TrmK and/or tRNA would cause some conformational changes upon tRNA binding to maintain the proper distance and orientation between A22 and SAM for the methyl transfer reaction. Our model is basically the same as the previous tRNA–TrmK complex model predicted by Dégut *et al* (48).

## Discussion

Transfer RNA undergoes multiple posttranscriptional modifications that play important roles in fine-tuning the decoding system and adapting the secondary and tertiary structures. tRNA modifications are also related to a myriad of cellular processes such as regulation of the metabolome, immune response, formation of viral particles, and gene silencing (16, 64, 65). Loss of tRNA modification results in severe diseases in humans such as mitochondrial disease (MELAS), heart disease, and neurological disorders (21, 66). Thus, understanding how tRNAs are modified by tRNA modification enzymes is critical. To address this issue, we applied high-throughput sequencing technology to functional analyses of tRNA modification enzyme and utilized tRNA-MaP to analyze tRNA–protein interactions.

While our method detected various natural modifications such as s^4^U8, m^1^A22, and anticodon modifications, the technique was not able to detect silent modifications such as dihydrouridine and pseudouridine (Fig. 1*B*). This issue could be addressed by taking advantage of chemical agents such as sodium borohydride (NaBH_4_) and rhodamine for dihydrouridine and 1-cyclohexyl-(2-morpholinoethyl) carbodiimide metho-p-toluene sulfonate (CMCT) for pseudouridine (67, 68) and coupling them to tRNA-MaP in the future. Recent studies show that the modification levels in tRNA are varied upon culture conditions and/or cellular stress in both prokaryotes and eukaryotes (12, 40, 69). The regulation of anticodon modification levels has a huge impact on the anticodon stem and loop structure and causes selective translation for stress response (69, 70). For example, the formation of the t^6^A modification at position 37 is catalyzed by multiple proteins including TsaC, TsaC1, and the TsaB–TsaE–TsaD complex in bacteria (71). The t^6^A37 acts as a positive determinant for isoleucyl-tRNA synthetase and prevents frame-shifting errors during translation (72). Thus, t^6^A37 plays a central role in translation. Based on our protein orthology analysis, these proteins are also conserved in *G. stearothermophilus* (Fig. S2). If *G. stearothermophilus* has the same anticodon modification pattern with *B. subtilis*, tRNA^Arg_CCU^, tRNA^Ile_CAU^, tRNA^Ser_GCU^, and tRNA^Thr_UGU^ should possess the t^6^A modification. However, unexpectedly, we did not detect the mutation signal from the t^6^A37 modification in these native tRNAs (Fig. 2*B*). The t^6^A modification level in these tRNAs under our culture conditions might be too low to be detected. We showed that tRNA-MaP is able to quantify relative modification levels in tRNA with a sensitivity similar to filter assays (Fig. 4*B*). Thus, tRNA-MaP is useful to quantify the relative modification levels of multiple tRNAs from various samples, which would provide key mechanistic insights into how tRNA modification levels adapt upon changes of environmental conditions such as heat stress, oxidative stress, starvation, etc.

The tRNA modifications of the three-dimensional core in tRNA generally enhance tRNA stability. For example, s^4^U8 increases the thermostability in tRNA by up to 5 °C (73). Also, the combination of Gm18, m^5^s^2^U54, and m^1^A58 in tRNA is known to increase tRNA stability (74). In contrast, while m^1^A22 is located in the D-arm region, the effect of the m^1^A22 modification on tRNA thermostability is unknown. The crystal structure of *T. thermophilus* tRNA^Leu^ shows multiple hydrogen-bonding networks and a base-stacking effect around A22 (Fig. S7*C*). We hypothesize that the methyl group in m^1^A22 could enhance the base-stacking effect *via* hydrophobic interactions, which may contribute to the stabilization of the tRNA structure. To address this, genetic and thermodynamic experiments are necessary.

To determine whether tRNA-MaP could be used to examine functional analyses of tRNA modification enzyme, we investigated *G. stearothermphilus* TrmK. We prepared 240 tRNA variants and successfully calculated the methyl group acceptance activity of all the variants using tRNA-MaP (Fig. 5). Our results showed that about 10 million reads were sufficient for the Seqkit analysis of 240 tRNA variants. The averaged read number per variant is calculated to be 41,666 (=10,000,000 [reads]/240 [variant] = 41,666 [reads/variant]). Thus, in theory, using the Illumina NextSeq 550 platform with the high-output kit that yields up to 400 million reads would allow us to analyze at least 5000 RNA variants (= 400,000,000 [reads]/41,666 [reads/variant]/2 [plus/minus enzyme reaction]) in a single sequencing experiment. Although this does not cover all double variants (240 × 239 = 57,360 variants), there is still enough room to test a number of double variants or tRNAs with multiple variations. tRNA-MaP with such variants reveals whether covariations restore the methyl group acceptance activity and how disruption of RNA secondary/tertiary structure affect the activity. Thus, the data would provide more detailed tRNA recognition mechanism of tRNA modification enzymes.

The tRNA modification profiles of *B. subtilis* and *G. stearothermophilus* are known to have slight differences. For example, *B. subtilis* tRNA^Leu^ possesses m^1^A58 (75), whereas *G. stearothermophilus* tRNA^Leu^ does not. In fact, *G. stearothermophilus* does not encode a tRNA (m^1^A58) methyltransferase (TrmI) homolog gene as shown in our orthology analysis (Table S1). The TrmK protein is, however, conserved in both *B. subtilis* and *G. stearothermophilus*. In the pioneer study, Dégut *et al.* reported structural and functional studies of *B. subtilis* TrmK wherein they solved the crystal structure of *B. subtilis* TrmK and performed extensive biochemical analyses using various tRNA transcripts and proposed a TrmK-tRNA docking model based on their biochemical and NMR studies (48). They showed that TrmK methylates seven tRNA species (tRNA^Ser^, tRNA^His^, tRNA^Tyr^, tRNA^Cys^, tRNA^Gln^, tRNA^Glu^, and tRNA^Leu^) (48). Also, they showed that purine at position 13 that forms a non-Watson-Crick base pairing with A22 is important for the TrmK reaction. This was concluded by restoration of the methyl group acceptance activity in the nonsubstrate tRNA^Gly^ by U13G variation (48). Furthermore, comparison of the methyl transfer activity in TrmK with native *E. coli* tRNA^Ser^ and tRNA^Ser^ transcript revealed that natural tRNA modifications restrict the TrmK reaction (48). Their major conclusion on the substrate recognition by TrmK is that TrmK requires full-length tRNAs with the L-shaped tRNA structure with purine 13 and A22 (48). In this study, we succeeded reproducing their results using *G. stearothermophilus* TrmK and added some updates on the enzymatic properties. First, our *in vitro* tRNA-MaP analysis confirmed that TrmK methylates the seven tRNAs (Fig. 3). Furthermore, our *in vivo* tRNA-MaP analysis showed that TrmK methylates five tRNA^Ser^ isoacceptors, five tRNA^Leu^ isoacceptors, and three tRNA^Gln^ isoacceptors (Fig. 1*B*). Second, our biochemical assays showed that TrmK methylates tRNA^Ser^, tRNA^Glu^, and tRNA^Gly^ U13A transcripts (Fig. 7). In particular, the sequence of *G. stearothermophilus* tRNA^Gly^ is identical with the sequence of *B. subtilis* tRNA^Gly^. While tRNA^Gly^ WT is not methylated by *B. subtilis* TrmK, the U13G variation restores the activity (48). This was also reproduced with *G. stearothermophilus* TrmK and tRNA^Gly^ U13A (Fig. 7*F*). Finally, our biochemical data suggested that TrmK recognized U8, A14, G18, and U55 bases in addition to the L-shaped structure. These results suggest that the tRNA recognition mechanism of *B. subtilis* TrmK and *G. stearothermophilus* TrmK is thought to be quite similar. During the preparation of this article, crystal structures of *Staphylococcus aureus* TrmK complexed with AdoMet and AdoHcy have been reported (76). The structure of *S. aureus* TrmK is also similar to *B. subtilis* TrmK, and thus the TrmK enzymes from the three organisms are thought to have a similar mechanism of substrate recognition.

We have proposed a model for the TrmK–tRNA complex using the data from tRNA-MaP and other biochemical assays (Fig. 8*B*). In the model, the distance between the N1 atom in A22 and the sulfur atom in S-adenosyl-L-homocysteine (AdoHcy) is 9.4 Å (Fig. S7, *A* and *B*). In addition, the distance of carbon bond between the reactive methyl group and the sulfur atom in AdoMet is 1.8 Å (PDB:1RG9) (77). This led us to estimate the distance of the N1 atom in A22 and methyl group in AdoMet to be roughly ∼7.6 Å. Thus, some conformational changes of TrmK and/or tRNA are required for the methyl-transfer reaction. The previous study of *B. subtilis* TrmK suggested that the Watson-Crick base pairing between U13 and A22 in tRNA causes loss of the methyl group acceptance activity in the tRNAs (48). Our experiment using *G. stearothermophilus* TrmK showed the same results. The Watson-Crick base pair in U13-A22 changes the orientation and distance of the N1 atom from the catalytic pocket. Thus, due to the U13-A22 basepairing, the adenine base needs to be flipped out, which is likely to reduce the reaction efficiency. By contrast, in the complex model, the N1 atom in A22 is orientated directly toward the methyl group donor. In addition, the orientation of the A22 residue is determined by multiple hydrogen-bonding networks (Fig. S7*C*). For example, G13-A22 and G12-C23 form the non-Watson-Crick base pair and Watson-Crick base pair, respectively (Fig. S7*C*). A22 interacts with C23 *via* basestacking (Fig. S7*C*). This probably explains why the variations that disrupt the D-stem, which would change the A22 orientation, drastically decreased the methyl group acceptance activity at position 22 (Fig. 5*B*).

We found that the U8, A14, G18, and U55 nucleotides are important for the tRNA recognition (Figs. 6 and 8*A*). The A14 and G18 are closely located on the TrmK surface in our complex model, suggesting that these bases could be directly recognized by TrmK. On the other hand, the structural elements U8 and U55 could be sensed by TrmK *via* structural changes in tRNA caused by U8 and U55 variations (Fig. 6). The variable region in tRNA^Leu^ forms a stem and loop structure (Fig. 5*H*). Our results show that the weakened stem in the variable region causes a significant drop in methyl group acceptance activity (Figs. 5*D* and 6*E*). We speculate that the variations in the variable region unfold the stem structure and result in a long-loop structure at 60 °C (reaction temperature), which could lead to a structural rearrangement of the three-dimensional core in tRNA or interfere with TrmK *via* steric hindrance. To fully address these points, structural analyses of the TrmK–tRNA complex are necessary. In sum, our study showed that the experimental restraints obtained from tRNA-MaP can be applied for structural modeling of a tRNA–protein complex. Using tRNA-MaP combined with the already-established assay methods, we provided the detailed tRNA recognition mechanism of TrmK where TrmK recognizes multiple nucleotides including U8, A14, G18, and U55 and prefers a compacted variable region (Fig. 8*A*).

Overall, tRNA sequencing technologies have been recently developed and improved, and thus have broad applications such as quantification of tRNA abundance, aminoacylation levels, and *in vivo*/*in vitro* tRNA structure. Our study has further diversified the technology as we show that tRNA-MaP can quantify relative levels of tRNA modifications and can be applied to the functional analyses of tRNA modification enzyme. tRNA-MaP performs the rapid screening of substrate tRNA for tRNA modification enzymes and identifies key residues for the substrate recognition using several hundreds of tRNA variants, which is not executable by filter and gel assays only. In theory, our technique is also applicable to any RNA molecules such as rRNA and microRNA that are also extensively modified, and even DNA molecules, which could accelerate the understanding of the biological properties of nucleic acids and nucleic acid–related proteins.

## Experimental procedures

### Strain, medium, and culture

*G. stearothermophilus* strain 10 (DMS13240/CIP106956) was purchased from Leibniz Institute DSMZ-German Collection of Microorganisms and Cell Cultures GmbH and was precultured in 50 ml of Medium 92 (pH 7.0) containing 1.5 g of trypticase soy broth (MP Biomedicals), 0.15 g of yeast extract (Nacalai Tesque, INC.) at 55 °C overnight. Forty milliliters of the precultured cells were inoculated into 400 ml of fresh Medium 92 and cultured at 55 °C for 8 h.

### Preparation of tRNA fraction

Total RNA was extracted by the acid guanidium thiocyanate-phenol-chloroform method as described in the literature (10, 78, 79). The tRNA fraction was purified by 10% PAGE containing 7 M urea, eluted from the gel in TEN250 buffer [10 mM Tris–HCl (pH 7.6), 1 mM EDTA, and 250 mM NaCl] at 4 °C overnight, and recovered by ethanol precipitation.

### Library preparation

The library preparation is composed of four steps (Fig. 1*A*).

First step: Deacylation and dephosphorylation of RNA: Ten micrograms of the tRNAs were deacylated in 100 μl of 100 mM Tris–HCl (pH 9.0) at 37 °C for 1 h and were recovered by ethanol precipitation. The tRNA (10 μg) was dissolved in 8 μl of water and then 1 μl of 10× CutSmart buffer (New England Biolabs) and 1 μl of Shrimp Alkaline Phosphatase (rSAP, 1 unit; New England Biolabs, code: M0371S) were added. The reaction mixture was incubated at 37 °C for 1 h.

Second step: Adapter ligation: Eighty picomoles of adapter DNA (5′-pCTG TAG GCA CCA TCA ATddC-3’; ddC, dideoxycytidine) (Integrated DNA Technology) were adenylated using a 5′DNA Adenylation Kit (New England Biolabs, code E2610S) at 65 °C for 1 h. The adapter ligation was then carried out in a 20 μl reaction mixture containing 1× T4 RNA ligase buffer (New England Biolabs), 25% PEG8000, 1 μg tRNA, 6 μM adenylated adapter, and 200 units T4 RNA ligase2 Truncated (New England Biolabs, code M0242S) at 16 °C for 16 h. The ligated tRNAs were purified on a 10% denaturing PAGE gel (7 M urea) and recovered by ethanol precipitation. The pellet was dissolved in 10 μl of water.

Third step: RT: The RT reaction was performed in a 10 μl reaction containing 50 mM Tris–HCl (pH 8.3), 75 mM KCl, 3 mM MgCl_2_, 1.25 mM dNTPs, 5 mM DTT, 1.25 μM RT primer, 1 μl of the ligated tRNA, and 100 units of TGIRT-III enzyme (Thermostable Group II intron reverse transcriptase, InGex, LLC) at 42 °C for 16 h. The RT primer used was 5′-pAGA TCG GAA GAG CGT CGT GTA GGG AAA GAG TGT/iSp18/CAA GCA GAA GAC GGC ATA CGA GAT ATT GAT GGT GCC TAC AG-3′ where ‘iSp18’ is an internal 18-atom Hexa-ethylene glycol spacer. After the reaction, 0.5 μl of 2 M NaOH was added to the reaction mixture, and then the template RNA was heat-degraded at 95 °C for 5 min. The cDNA was separated on a 10% denaturing PAGE gel (7 M urea), purified by gel extraction, and recovered by ethanol precipitation. The cDNA was dissolved in 10 μl of water.

Fourth step: Circularization of cDNA and indexing PCR: The cDNA was then circularized in a 15 μl reaction containing 1× reaction buffer (Lucigen), 2.5 mM MnCl_2_, 1 M Betaine, 5 μl of cDNA, and 50 units of Circligase II (ssDNA ligase, Lucigen) at 60 °C for 2 h. The circularized cDNA was amplified in a 75 μl PCR reaction mixture containing 1× buffer for KOD-Plus-Neo (Toyobo), 1.5 mM MgSO_4_, 0.3 μM forward primer, 0.3 μM reverse indexing primer, 0.2 mM dNTPs, 9 μl of the circularized cDNA, and 1.5 units KOD-Plus-Neo (Toyobo) for 11 to 17 cycles depending on the cDNA concentration. The PCR program used was 98 °C for 30 s; 11 to 17 cycles of 98 °C for 10 s, 60 °C for 10 s, 68 °C for 15 s; 68 °C for 5 min; 4 °C hold. The PCR product was separated by an 8% nondenaturing PAGE gel in 1× TBE buffer at 180 V for 1 h, extracted from the gel, and recovered by NucleoSpin Gel & PCR Clean-up (Macherey-Nagel). The quality check of the library was checked on an Agilent 2100 Bioanalyzer system (Agilent) and ABI 7500 Realtime PCR system. The library was sequenced by Illumina NextSeq (35% PhiX, 150 cycles, single-end sequencing, Mid-output flow cells) or Illumina MiSeq (15% PhiX, 150 cycles, single-end sequencing, MiSeq reagent kit v3) with the standard Illumina sequencing primer. Each oligonucleotide used in the library preparation is summarized in Table S3.

### Data analysis

The tRNA genes encoded in the *G. stearothermophilus* genome were predicted by tRNAscan-SE-2.0 (80). The software predicted 90 tRNA genes. Of these, the genes encoding identical tRNA sequences were computationally combined. The tRNA genes that scored less than 70 were filtered out, which resulted in reference genes for 59 tRNA sequences (Table S4). The CCA-coding region was added to the 3′-end of those tRNA genes that lack the CCA terminus.

The 3′ adapter sequence was removed by Cutadapt (81). ShapeMapper 2 (82) was used for the alignment to the reference tRNA genes and the calculation of mutation rates with the options of --min-seq-depth 1000 and --indiv-norm where native tRNA samples were input as “modified sample” and tRNA transcript samples were input as “untreated sample” to profile the natural modifications in *G. stearothermophilus* tRNA. For the m^1^A22 profiling experiments, tRNA transcripts methylated by TrmK were input as “modified sample” and tRNA transcript samples were input as “untreated sample”. Alternatively, the mutation rates at A22 were also calculated where the read number of a specified sequence that has mutations in A22 was directly counted with SeqKit (57). The mutation rate at position 22 (M_rate_) was defined as

$$M_{rate}=\frac{Read_{Mutant}\;}{Read_{Total}\;}$$

where Read_Mutant_ is the read number from the sequences that possess a point mutation at position 22 in a specified tRNA (*e.g*., sum of the read number from A22G, A22C, and A22U in tRNA^Leu^-GAG-2) and Read_Total_ is the total read number in that tRNA (*e.g.* sum of the read number from WT, A22G, A22C, and A22U in tRNA^Leu-GAG-2^). M_rate_ (modified sample) and M_rate_ (untreated samples) were calculated independently and then, the final M_rate_ was calculated by subtraction of M_rate_ (untreated sample) from M_rate_ (modified sample).

The differential expression analysis was performed as shown in the literature (40). In brief, low quality reads in the adapter-trimmed fastq files were removed by Trimmomatic (83). Then, tRNA abundance was estimated by Salmon (84) and analyzed by DESeq2 (85). The data from the native tRNA pool from *G. stearothermophilus* and the synthetic tRNA pool from T7 RNA polymerase transcription was visualized as a volcano plot as shown in Fig. S5.

### Ortholog analysis

All protein sequences of *B. subtilis* sp. subtilis strain 168 (4237 protein sequences) and *G. stearothermophilus* strain 10 (3304 protein sequences) were retrieved from NCBI. All proteins of *G. stearothermophilus* were queried against all proteins of *B. subtilis*, which provided 2068 orthologous proteins scored an e-value < 1 × 10^-20^. Then, a list of tRNA modification enzymes in *B. subtilis* was obtained from the reference (27) to identify the tRNA modification enzymes in *G. stearothermophilus*. Several tRNA modification enzymes found in *E. coli* K-12 and *T. thermophilus* were also examined. The BLAST results are summarized in Table S1.

### Preparation of *G. stearothermophilus* tRNA transcripts

The *G. stearothermophilus* tRNA transcripts were prepared by *in vitro* T7 RNA polymerase transcription. To improve the transcription efficiency, an extra G was added to the 5′-end of those tRNAs that do not start with a G. The oligonucleotide pools of the tRNA mixture were designed in the following cassette: 5′-**GC GTA ATA CGA CTC ACT ATA** (tRNA sequence) TGA GAC CGG ATC CGG ATC CCC GCT GAG CAA TAA CTA GC-3′, where the bold region is the T7 promoter, the underlined region is the T7 terminator, the (tRNA sequence) is a DNA template for tRNA, and the dash under-lined region is the cleavage site of BsaI. The oligonucleotide pools were purchased from Integrated DNA Technology or Twist Bioscience HQ. The oligonucleotide pools were amplified by PCR using a primer set of 5′-GCG TAA TAC GAC TCA CTA TA-3’ (forward primer) and 5′-GCT AGT TAT TGC TCA GCG G-3’ (reverse primer). All oligonucleotides used in this study are provided in Table S3. The PCR product was purified by phenol extraction and recovered by ethanol precipitation. The resultant DNA was dissolved in 100 μl of water. Forty micrograms of the DNA were digested in a 200 μl reaction mixture containing 1× CutSmart buffer (New England Biolabs) and 240 units BsaI-HF v2 (New England Biolabs) at 37 °C for 16 h. The digested product was purified by 8% nondenaturing PAGE and recovered by ethanol precipitation. The DNA was dissolved in 200 μl of water. Transcription was performed in a 400 μl reaction mixture containing 40 mM Hepes-KOH (pH7.6), 5 mM DTT, 20 mM MgCl_2_, 1 mM spermidine, 20 μg bovine serum albumin, 2.5 mM NTPs, 200 μl template DNA, and 40 μl T7 RNA polymerase (made in house) at 37 °C for 4 to 6 h. The mixture of tRNA transcripts was purified using a 10% denaturing PAGE gel (7 M urea) and recovered by ethanol precipitation.

### Cloning, expression, and purification of *G. stearothermophilus* TrmK

The genomic DNA of *G. stearothermophilus* JCM14450 was purchased from RIKEN BioResource Research Center. The primer set of 5′-CCG CGC GGC AGC CAT ATG AAC GAG TTT CGC CTA TCA AAG CGG T-3’ (forward primer) and 5′-GTG GTG GTG CTC GAG TCA TCG CAA CGC CTC CTC TAC CAA TTG GAC-3’ (reverse primer) corresponding to the *trmK* region (LQIE01000144.1:47002-47706) were used for PCR amplification. The resulting *trmK* gene was cloned into a pET28a vector and expressed as a histidine-tagged protein in *E. coli* BL21 (DE3) Rosetta2 strain. The *E. coli* cells transformed with pET28a-*trmK* were grown in 250 ml of LB medium at 37 °C for 16 h and inoculated into 1 l of fresh LB medium and further grown at 37 °C. After 2 h cultivation, the TrmK expression was induced by the addition of 1 mM IPTG at 20 °C for 24 h. Two grams of the cells were resuspended in 10 ml of buffer A containing 50 mM Tris–HCl (pH 8.0), 500 mM NaCl, 1× protease inhibitor cocktail (Nacalai Tesque), and 5% glycerol and sonicated with an ultrasonic disruptor (model VCX-500, Sonics and Materials. Inc) on ice for 10 min. After centrifugation at 10,000*g* at 4 °C for 15 min, the supernatant was filtered with a 0.22 μm syringe filter and then loaded onto a HisTrap HP (5 ml; Cytiva) column pre-equilibrated with 25 ml of buffer A. The unbound proteins were washed out with 25 ml of buffer A. The bound protein was eluted with a linear gradient of imidazole from 0 mM to 500 mM in buffer A. The TrmK fraction was dialyzed in buffer B containing 50 mM Tris–HCl (pH8.0), 200 mM NaCl, and 5% glycerol and passed through a Hitrap Q HP (5 ml; Cytiva) column to remove contaminating nucleic acids. The histidine tag was cleaved by thrombin (Millipore-Sigma) in a 500 μl reaction mixture containing 1× Thrombin buffer (Millipore-Sigma), 50 mM NaCl, 1 mg TrmK, and 10 units of thrombin at 4 °C for 16 h. The untagged TrmK was purified with Ni-NTA Fast Flow column chromatography. The TrmK was concentrated to 2 mg/ml using Vivaspin 15R centrifugal filter units (Sartorius Stedim Biotech). The purified TrmK was stored in a storage buffer containing 25 mM Tris–HCl (pH8.0), 100 mM NaCl, 2.5 mM MgCl_2_, 3 mM 2-mercaptethanol, and 50% glycerol at −30 °C.

### Measurement of TrmK activity

The enzymatic activity of TrmK was measured by a conventional filter assay and nucleoside analysis. For the filter assay, the TrmK reaction was performed in a 100 μl reaction mixture containing 50 mM Tris–HCl (pH 7.6), 200 mM KCl, 5 mM MgCl_2_, 2 μM tRNA, 10 μM AdoMet (a mixture of nonradioisotope and ^3^H-labeled AdoMet), and 100 nM TrmK at 60 °C. Fifteen microliters of the reaction mixture were taken at specific time points and spotted onto a filter paper (Whatman). The filter was washed with 5% trichloroacetic acid ten times at 4 °C for 5 min. The methyl group incorporation was measured by a liquid scintillation counter. The filter assay was independently replicated three times (n = 3). For the tRNA-MaP experiments, the TrmK reactions were performed in 500 μl reaction containing 50 mM Tris–HCl (pH 7.6), 200 mM KCl, 5 mM MgCl_2_, 2 μM tRNA, 10 μM AdoMet, and 100 nM TrmK at 60 °C for 10 min. The tRNAs were purified by phenol extraction and recovered by ethanol precipitation. The resulted tRNAs were used for library preparation. For nucleoside analysis, tRNAs were methylated in a 50 μl reaction mixture containing 50 mM Tris–HCl (pH 7.6), 200 mM KCl, 5 mM MgCl_2_, 6 mM 2-mercaptoethanol, 10 μM tRNA, 50 μM AdoMet, and 1 μM TrmK at 60 °C for 1 h. The tRNA was purified by phenol extraction and recovered by ethanol precipitation. The recovered tRNA was digested to nucleosides and analyzed by an HPLC system as described previously (10). For the gel assays, the TrmK reaction was performed in 16 μl reaction mixture containing 50 mM Tris–HCl (pH 7.6), 200 mM KCl, 5 mM MgCl_2_, 17 μM tRNA transcript, 50 μM ^14^C-AdoMet, and 100 nM TrmK at 60 °C for 10 min. After the reaction, 16 μl of 2× loading dye containing 0.02% brome phenol blue, 0.02% xylene cyanol, 7 M urea, and 1× TBE were added, and then 10 μl of the mixture was loaded onto a 10% polyacrylamide gel containing 7 M urea. After the electrophoresis, the tRNA transcripts were visualized by methylene blue staining. Incorporations of ^14^C-methyl group into the tRNA transcripts were monitored with a FLA-2000 imaging analyzer (GE Healthcare).

### Docking model of TrmK and tRNA

The TrmK–tRNA complex structure was modeled with HDOCK (63). The crystal structures of TrmK from *B. subtilis* (PDB: 6Q56) and tRNA^Leu^ from *T. thermophilus* (PDB: 2BYT) were used for the modeling with the experimental restraints in which receptor-binding site residues (5:A, 29:A, 200:A, 220:A, 227:A) and ligand-binding site residues (7-8:B, 10-15:B, 18-19:B, 20:B, 23-29:B, 31:B, 41:B, 43-44:B, 49:B, 52-53:B, 55-66:B, 68-72:B) were used. The top-scoring complex structure was visualized with PyMol. Then, the crystal structure of *M. capricolum* TrmK complexed with AdoHcy (PDB: 6QE6) (86) was superposed with the complex structure with an RMSD value of 1.208, to model the AdoHcy-binding site.

## Data availability

All sequencing data used in this study are available at DNA Data Bank of Japan (DDBJ) Sequence Read Archive (SRA) under the accession numbers of DRR379694-DRR379707. All data used in this study are available from the corresponding authors upon request.

## Supporting information

This article contains supporting information.

## Conflict of interest

The authors declare no conflict of interest with the contents of this article.
