# Supplementary material for: Application of mutational profiling: New functional analyses reveal the tRNA recognition mechanism of tRNA m1A22 methyltransferase
Source: J Biol Chem. 2022 Dec 1;299(1):102759. doi: 10.1016/j.jbc.2022.102759 (PMC9801127; doi:10.1016/j.jbc.2022.102759)
Supplement: Supporting information [file mmc1.docx]

**Supporting Information**

**Application of mutational profiling: new functional analyses reveal the tRNA recognition mechanism of tRNA m^1^A22 methyltransferase (TrmK)**

Ryota Yamagami^*^, and Hiroyuki Hori^*^

Department of Materials Science and Biotechnology, Graduate School of Science and Engineering, Ehime University, 3 Bunkyo-cho, Matsuyama, Ehime 790-8577, Japan

* To whom correspondence should be addressed.

Hiroyuki Hori

Tel: +81-89-927-8548

Fax: +81-89-927-8548

Email: hori.hiroyuki.my@ehime-u.ac.jp

Address: 401 Engineering Bldg 3, 3 Bunkyo-cho, Matsuyama, Ehime 790-8577, Japan

Ryota Yamagami

Tel: +81-89-927-9919

Fax: +81-89-927-9919

Email: yamagami.ryota.bn@ehime-u.ac.jp

Address: 410 Engineering Bldg 3, 3 Bunkyo-cho, Matsuyama, Ehime 790-8577, Japan

**Supplementary Figures**


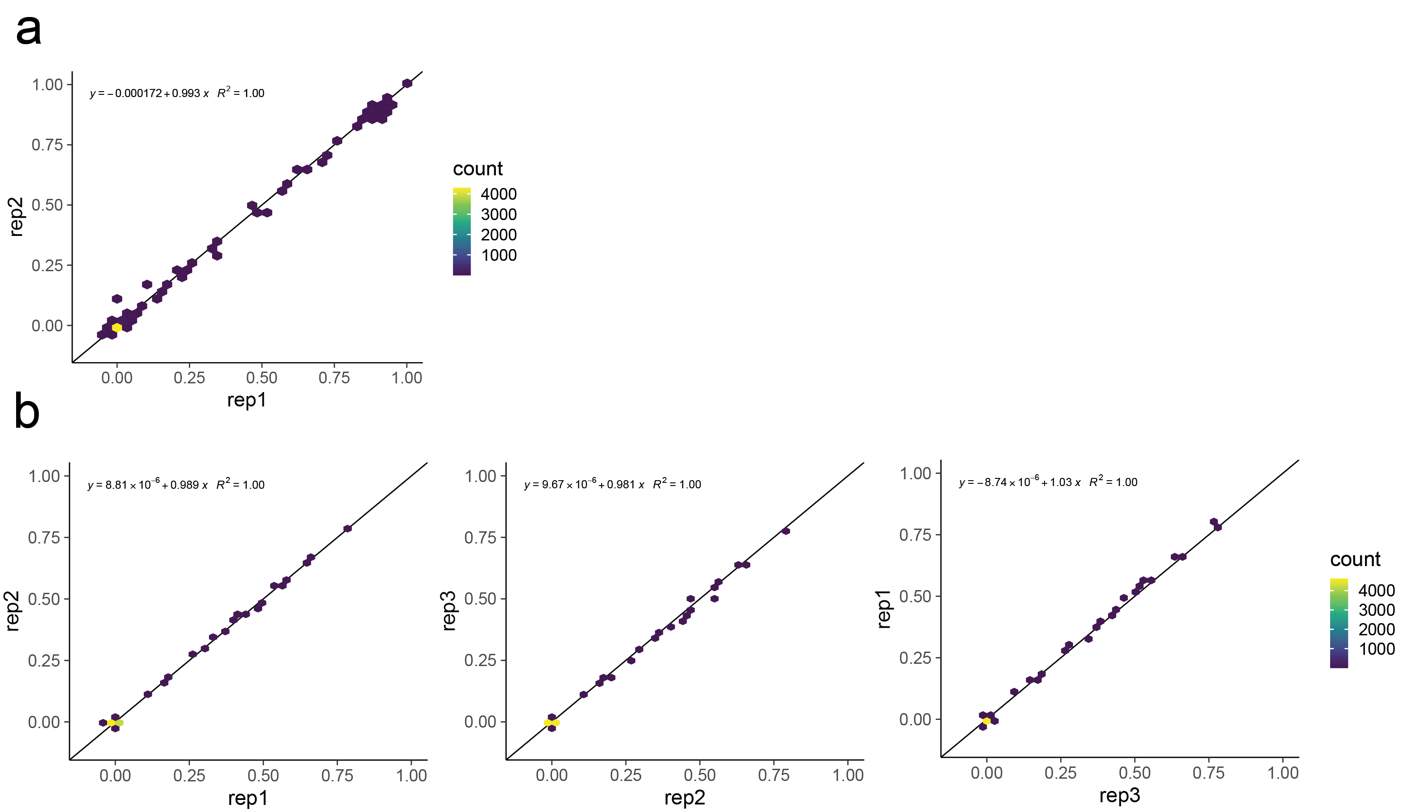


**Figure S1 | tRNA-MaP provides reproducible data.** **(a - b)** The mutation rates between each replicate for (a) *G. stearothermophilus* native tRNAs and (b) *G. stearothermophilus* tRNA transcripts are plotted.


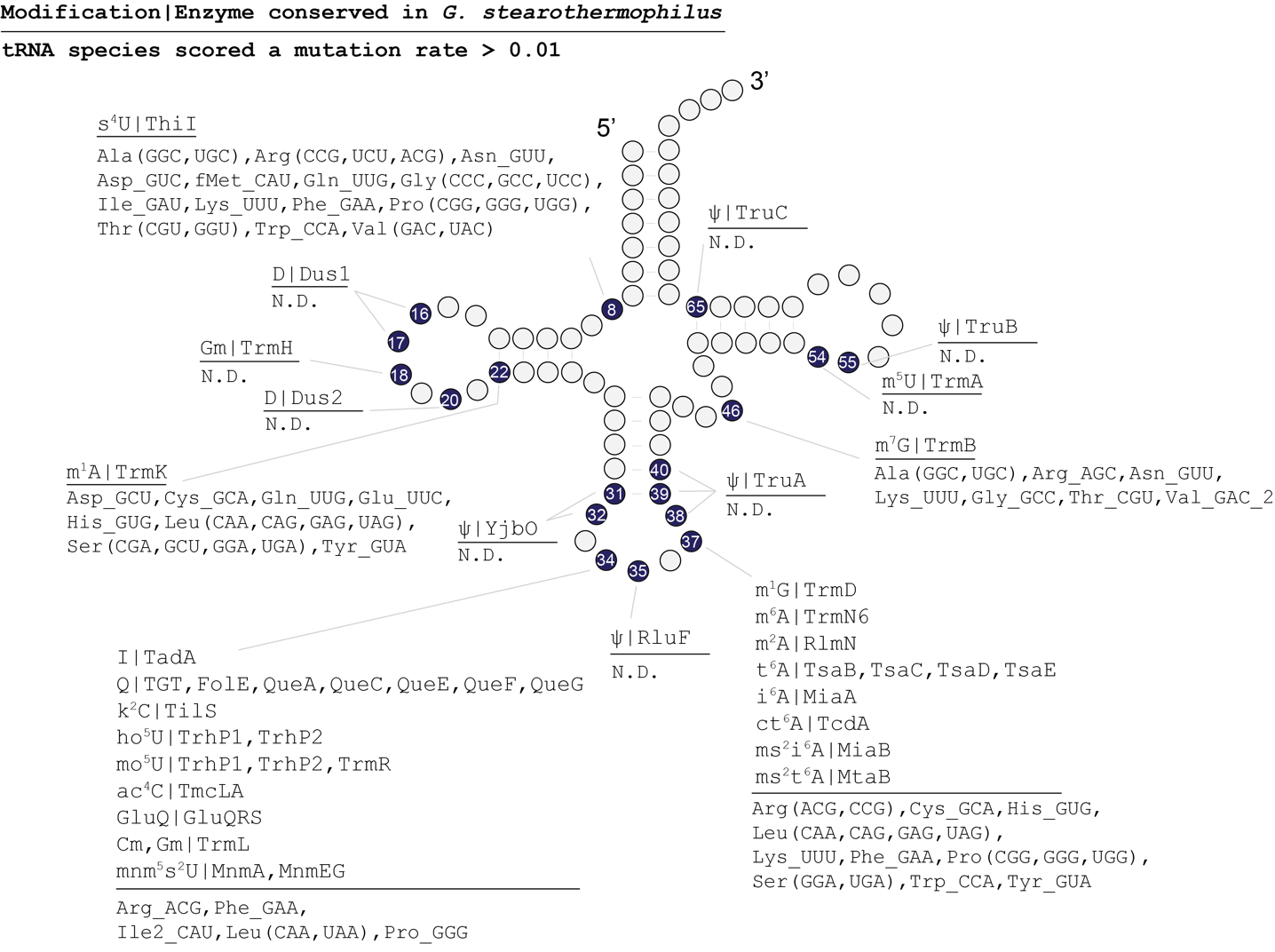


**Figure S2 | Protein orthology and tRNA-MaP analyses predict provisional tRNA modifications in *G. stearothermophilus* tRNAs.** Orthologous proteins of *B. subtilis* conserved in *G. strearothermophilus* with an e-value less than 1E-20 retrieved by a blastp search are mapped onto the secondary structure of tRNA. The tRNA species that scored mutation rate > 0.01 at the modification sites are also shown.

**
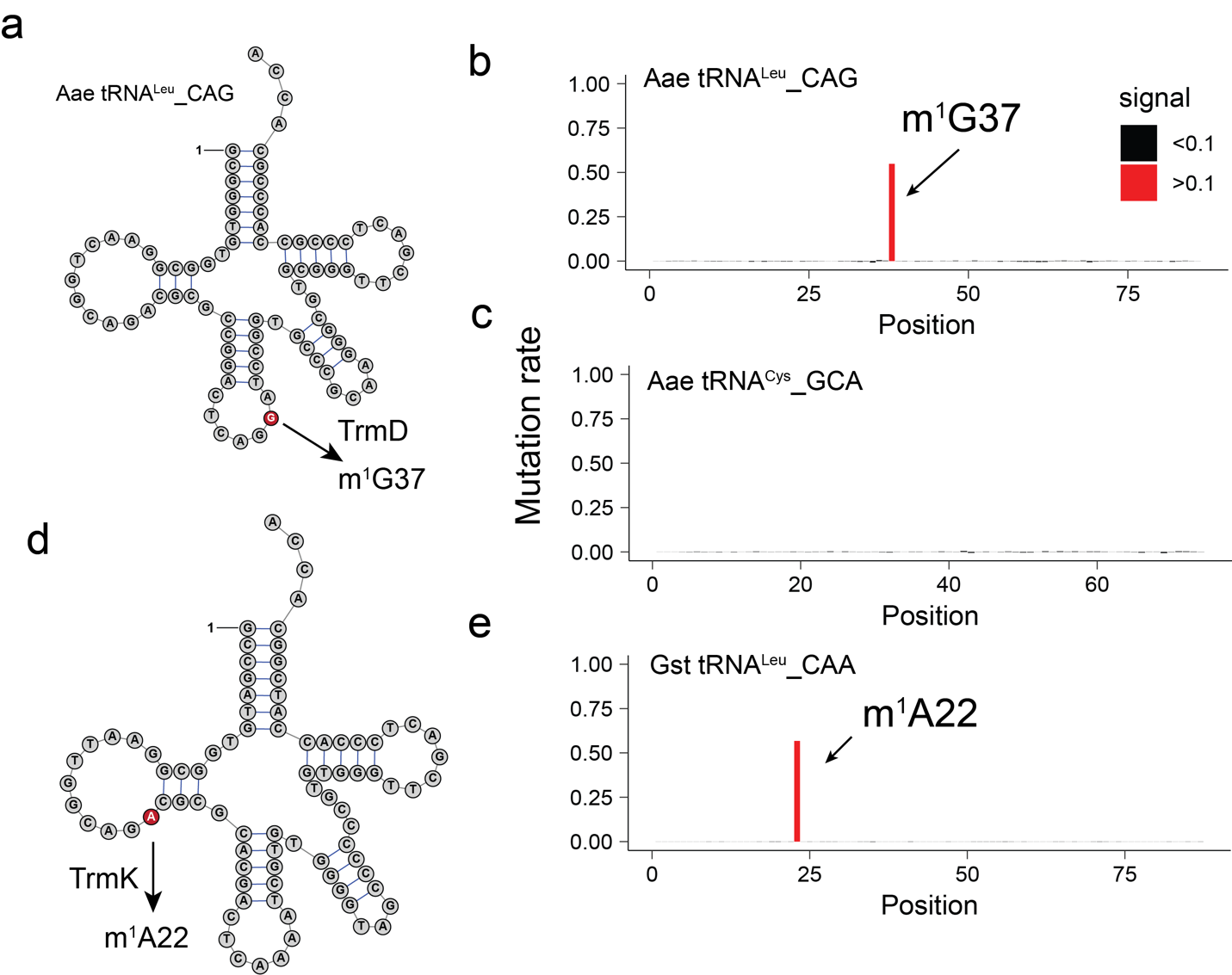
**

**Figure S3 | tRNA-MaP detects m^1^G37 and m^1^A22.** **(a)** The secondary structure of A. aeolicus tRNA^Leu^. The G nucleotide at position 37 is methylated by the m^1^G37 methyltransferase (TrmD). **(b - c)** tRNA-MaP detects the mutation signal from m^1^G37 in (b) tRNA^Leu^ (a substrate tRNA for TrmD) but not in (b) tRNA^Cys^ (non-substrate tRNA for TrmD). **(d)** The secondary structure of *G. stearothermophilus* tRNA^Leu^. The Adenosine at position 22 is methylated by the m^1^A22 methyltransferase (TrmK). **(e)** tRNA-MaP detects the mutation signal from m^1^A22 in the tRNA^Leu^.


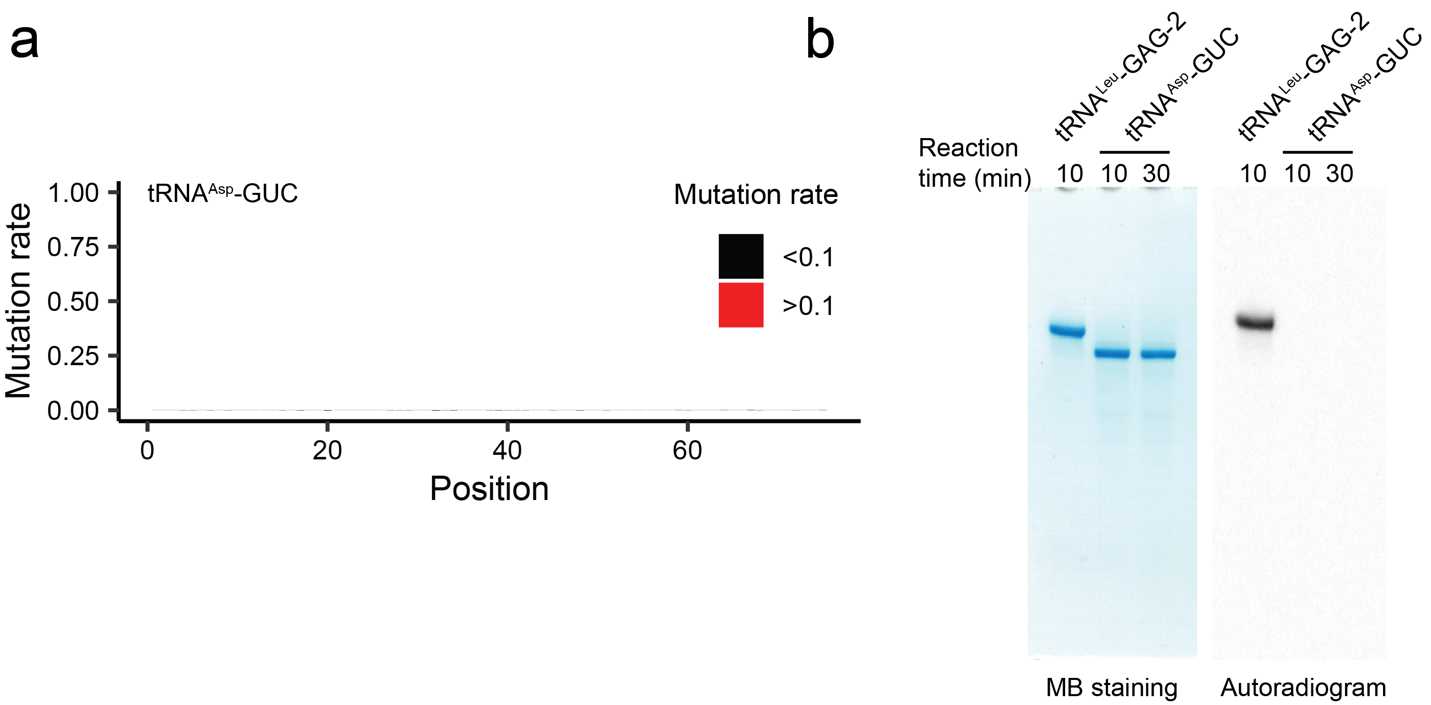


**Figure S4 | tRNA^Asp^ does not have the methyl group acceptance activity.** **(a)** tRNA-MaP does not detect the mutation signal at A22 in the tRNA^Asp^ transcript. **(b)** The methyl group acceptance activity in tRNA^Asp^ transcript was tested by a gel assay. The TrmK reaction was performed in the reaction mixture containing 50 mM Tris-HCl (pH 7.6), 200 mM KCl, 5 mM MgCl_2_, 0.0125 OD/µL tRNA, 50 µM ^14^C-S-adenosyl-L-methionine (AdoMet), and 100 nM TrmK at 60 ºC. The gel was stained with methylene blue (MB), and ^14^C-methyl group incorporation was monitored by autoradiography.


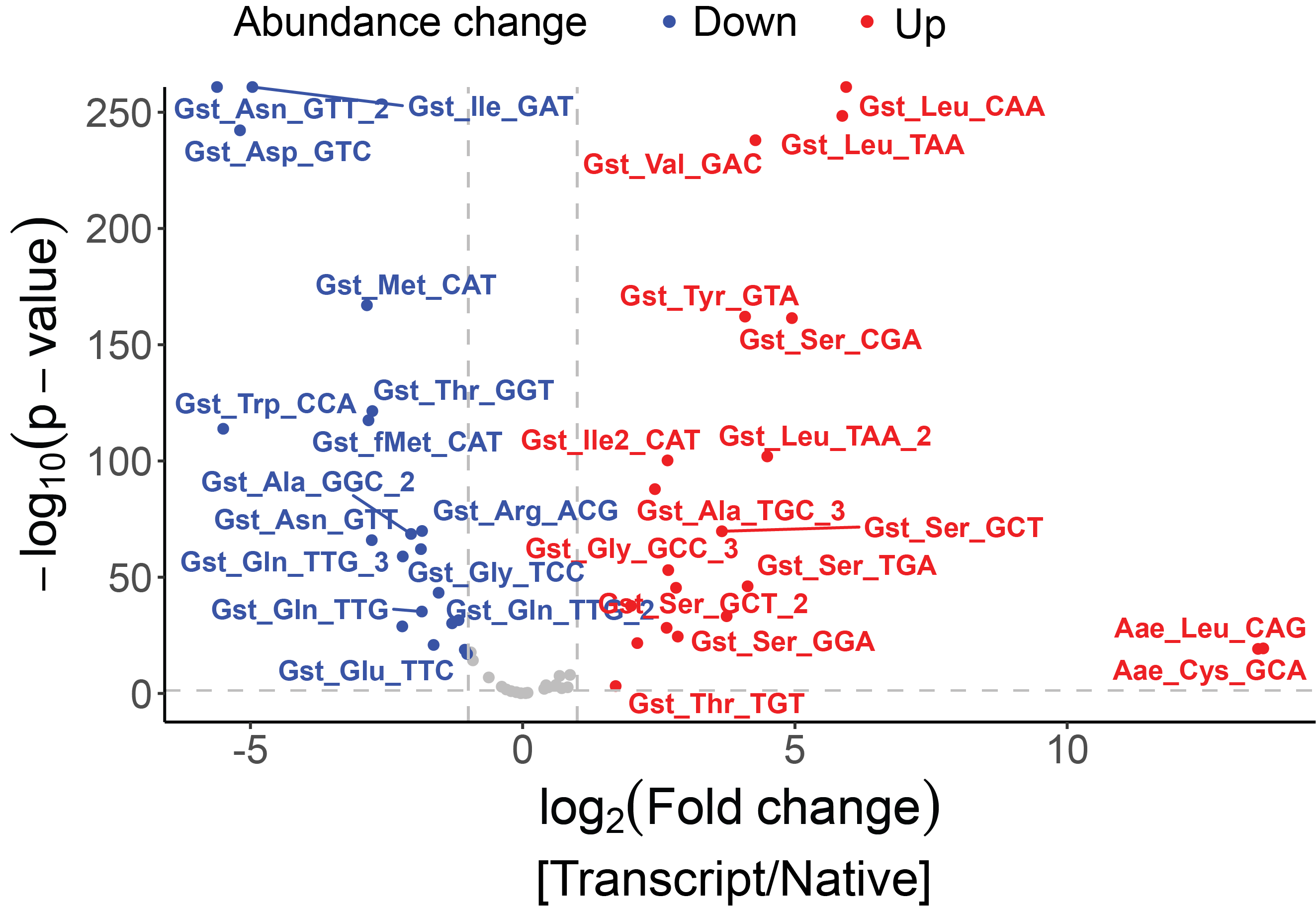


**Figure S5 | Comparison of tRNA abundance in native tRNA mixture and tRNA transcripts.** The differential expression analysis was performed using unmodified samples from the native tRNA mixture (n = 2) and tRNA transcripts (n = 3).


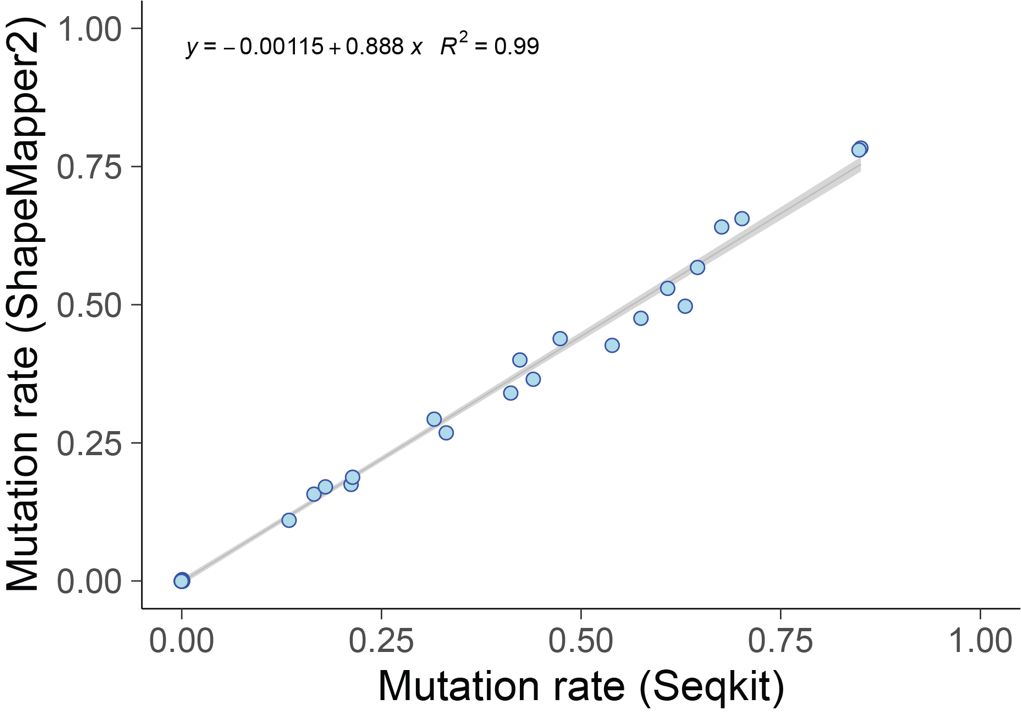


**Figure S6 | Mutation rates calculated with the Seqkit method are highly correlated with the rates calculated with ShapeMapper 2.** ShapeMapper 2 performs the variant calling with the alignment-based method where an alignment software (either Bowtie2 or STAR) is used. To avoid ambiguous calculation of the mutation rates, the number of reads that have a specified sequence was directly counted using Seqkit. The mutation rates at A22 in *G. stearothermophilus* tRNA transcripts (that have sequence diversity) were calculated by the Seqkit analysis and compared with the rates at A22 calculated with ShapeMapper2. The python script used in the Seqkit analysis is provided in the Supplementary codes section.

**
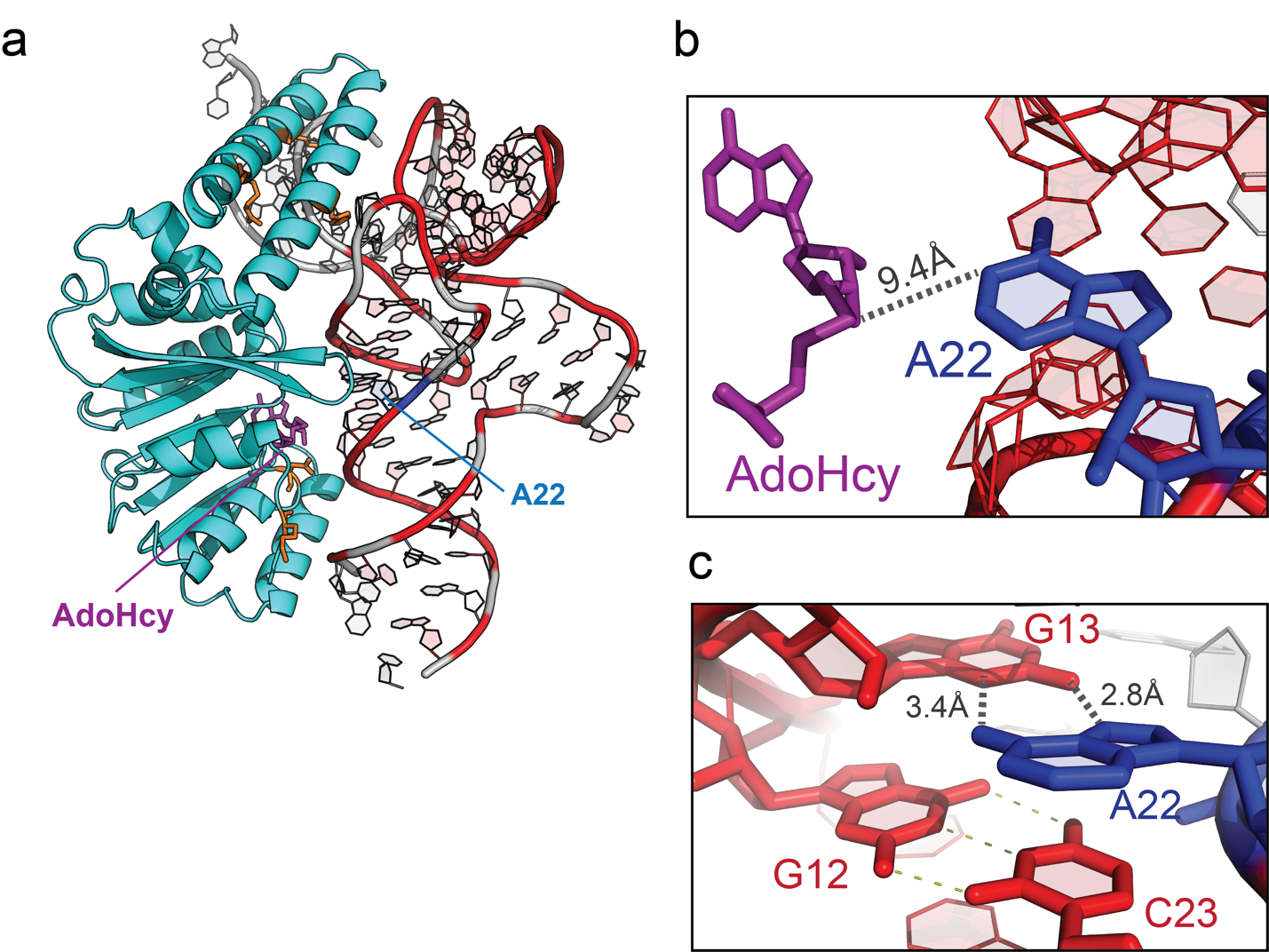
**

**Figure S7 | Structural elements in the TrmK-tRNA complex model. (a)** The distance between the A22 and AdoHcy binding site in the TrmK-tRNA complex model (see the main Figure 7) was measured. **(b)** The hydrogen bonding network around A22 is illustrated. The target A22 is highlighted in blue whereas the recognition sites of TrmK are highlighted in red.

**Supplementary codes**

A representative python script used in the Seqkit analysis was provided below.

1. #!/usr/bin/env python
2. # coding: utf-8
3. import subprocess
4. from subprocess import PIPE
5. import pandas as pd
6. import time
7. start_time=time.time()
8. def time_start():
9. global _start_time
10. start_time=time.time()
12. def time_finish():
13. t_sec=round(time.time()- start_time)
14. (t_min, t_sec)=divmod(t_sec, 60)
15. (t_hour, t_min)=divmod(t_min, 60)
16. print('Time Passed: {}hour:{}min:{}sec'.format(t_hour, t_min, t_sec))
18. time_start()
19. proc=subprocess.run('conda activate bioinfo', shell=True, stdout=PIPE, stderr=PIPE)
20. def replace_char_at_index(old_str, index, replacement):
21. new_str=old_str
22. if index < len(old_str):
23. new_str=old_str[0:index]+replacement+old_str[index+1:]
24. return new_str
25. def listToString(s):
26. str1=" "
27. return(str1.join(s))
28. reference_fasta = pd.read_csv('mutant_sequence.fa', header=None)
29. print(reference_fasta)
30. seq_name_list=[]
31. for i in range(0, len(reference_fasta), 2):
32. seq_name=reference_fasta.iloc[i, 0]
33. seq_name=seq_name.replace(">", "")
34. seq_name_list.append(seq_name)
36. seq_list=[]
37. for i in range(1, len(reference_fasta), 2):
38. RNA_sequence=reference_fasta.iloc[i, 0]
39. seq_list.append(RNA_sequence)
40. Combined_data=[]
41. for sequence, name in zip(seq_list, seq_name_list):
42. A22T=replace_char_at_index(sequence, 21, "T")
43. A22G=replace_char_at_index(sequence, 21, "G")
44. A22C=replace_char_at_index(sequence, 21, "C")
45. #(4) Count sequence with Seqkit in both plus/minus reaction
46. print("target_name: "+name)
47. print("target_sequence: "+sequence)
48. print("target_sequence_A22T: "+A22T)
49. print("target_sequence_A22G: "+A22G)
50. print("target_sequence_A22C: "+A22C)
51. data=[]
52. data=[name]
53. data.append(sequence)
54. analyzing_list=[]
55. analyzing_list=[sequence, A22T, A22G, A22C]
56. analyzing_name=[]
57. analyzing_name=["WT", "A22T", "A22G", "A22C"]
58. for var, name in zip(analyzing_list, analyzing_name):
59. #for Modified
60. argument="seqkit grep -s -i -p %s B_Cutadapted_quality_trim/7_mutant_plus_TrmK_rep1/DK21255_07_trim.fastq.gz | grep @NB -c" %(var)
61. print("argument_modified("+name+"): "+argument)
62. proc=subprocess.run(argument, shell=True, stdout=PIPE, stderr=PIPE)
63. output_modified=proc.stdout.decode('utf8')
64. output_modified.rstrip('\n')
65. print("Read number_modified("+name+"):"+output_modified)
66. data.append(int(output_modified))
67. print()
68. #for Unmodified
69. argument="seqkit grep -s -i -p %s B_Cutadapted_quality_trim/5_mutant_minus_TrmK_rep1/DK21255_05_trim.fastq.gz | grep @NB -c" %(var)
70. print("argument_unmodified("+name+"): "+argument)
71. proc=subprocess.run(argument, shell=True, stdout=PIPE, stderr=PIPE)
72. output_unmodified=proc.stdout.decode('utf8')
73. output_unmodified.rstrip('\n')
74. print("Read number_unmodified("+name+"):"+output_unmodified)
75. data.append(int(output_unmodified))
76. print()
77. try:
78. total_read_modified=int(data[2])+int(data[4])+int(data[6])+int(data[8])
79. Mrate_modified=(int(total_read_modified)-int(data[2]))/total_read_modified
80. except ZeroDivisionError:
81. Mrate_modified=0
82. try:
83. total_read_unmodified=int(data[3])+int(data[5])+int(data[7])+int(data[9])
84. Mrate_unmodified=(int(total_read_unmodified)-int(data[3]))/total_read_unmodified
85. except ZeroDivisionError:
86. Mrate_unmodified=0
87. Mrate=Mrate_modified-Mrate_unmodified
88. print(total_read_modified)
89. print(Mrate_modified)
90. print(total_read_unmodified)
91. print(Mrate_unmodified)
92. print(Mrate)
93. data.append(total_read_modified)
94. data.append(Mrate_modified)
95. data.append(total_read_unmodified)
96. data.append(Mrate_unmodified)
97. data.append(Mrate)
98. Combined_data.append(data)
99. print("-------------------Done!-------------------")

102. Compiled_df=pd.DataFrame(Combined_data, columns=["sequence_name", "sequence", "Read_modified_WT", "Read_unmodified_WT",
103. "Read_modified_A22T","Read_unmodified_A22T", "Read_modified_A22G","Read_unmodified_A22G",
104. "Read_modified_A22C","Read_unmodified_A22C", "total_read_modified",
105. "Mrate_modified", "total_read_unmodified", "Mrate_unmodified", "Mrate"])
106. print(Compiled_df)
107. Compiled_df.to_csv(r'B_Compiled_data_rep1.csv', index = False, header=True)
108. time_finish()
